# The effects of therapeutic virtual reality experience to promote mental well-being in older people living with physical disabilities in long-term care facilities

**DOI:** 10.1186/s13063-023-07592-7

**Published:** 2023-08-26

**Authors:** Rick Yiu Cho Kwan, Fowie Ng, Linda Chiu Wa Lam, Rebecca Choy Yung, Olive Shuk Kan Sin, Sally Chan

**Affiliations:** 1 School of Nursing, Tung Wah College, Hong Kong SAR, China; 2 School of Management, Tung Wah College, Hong Kong SAR, China; 3 Department of Psychiatry, Chinese University of Hong Kong, Hong Kong SAR, China; 4 Golden Age Foundation, Hong Kong SAR, China; 5 Board of Director Office, Pok Oi Hospital, Hong Kong SAR, China; 6 Tung Wah College, Hong Kong SAR, China

**Keywords:** Virtual reality, Mental well-being, Physical disabilities, Older people, Long-term care facilities

## Abstract

**Background:**

Mental well-being is poor in long-term care facilities (LTCF) residents. Physical disabilities, impaired social engagement, and environmental stress are also common in LTCF which exacerbate the decline of the mental well-being of older people living in LTCF. Protective elements, including nature-based, reminiscence, outdoor, and group activities, are known to be effective to promote the mental well-being of older people living in LTCF. However, limited by their physical disabilities and poor social support, older people living in LTCF are not likely to benefit from these effective measures. Virtual reality has been proven to be feasible to be environmentally unrestricted to providing LTCF residents with all protective elements promoting mental well-being. However, its effects on the mental well-being of LTCF residents living with physical disabilities are unclear.

**Methods:**

This study employs a single-blinded, two-parallel-group (intervention-to-control group ratio = 1:1), non-inferiority, randomized controlled trial. Eligible participants are aged 60 years or above, LTCF residents, and living with physical disabilities. The study will be conducted in LTCF. In the intervention group, participants will receive a 6-week VR experience program. In the control group, participants will receive the usual care provided by the LTCF. The primary outcome is mental well-being, as measured by World Health Organization Five Well-being Index at the time point of baseline (i.e., week 0) and after completion of the intervention (i.e., week 7). This study aims to recruit a total of 216 participants. Generalized estimating equations (GEE) will be used to examine the effects of the intervention.

**Trial registration:**

The trial has been registered at ClinicalTrials.gov (Identifier: NCT05818579), Registered on April 5, 2023. The latest version of the protocol was published online on 19 April 2023. All items come from the World Health Organization Trial Registration Data Set. This study has been approved by the Research Ethics Committee of Tung Wah College, Hong Kong (reference number: REC2023158). The findings will be disseminated in peer-reviewed journals, presented at international and local conferences with related themes, and shared in local media.

**Supplementary Information:**

The online version contains supplementary material available at 10.1186/s13063-023-07592-7.

## Introduction

### Background and rationale

Physical disabilities refer to a physical condition that limits a person’s capacity to participate in activities of daily living [1]. In the scenario that the family of older people living with physical disabilities are not able to take care of them, they usually end up living in long-term care facilities (LTCF). A study showed that the prevalence of physical disabilities is up to 62% [2]. Physical disabilities are also associated with a lower level of social engagement, including participation in social organizations, use of senior center activities, talks to friends, and even use of the internet [3]. Unsurprisingly, a systematic review showed that their mental well-being is poor and that the prevalence rates of loneliness and depression among older people living in LTCF are high (56.92.5% and 11–85.5%, respectively) [4]. Physical disabilities cause poorer mental well-being [5]. Social engagement is also positively associated with the quality of life in older people with physical disabilities [6]. The detrimental effect of lower social engagement in institutionalized older people living with physical disabilities on mental well-being and quality of life is further exacerbated by the social distancing policies during and after the COVID-19 pandemic [7, 8].

Mental well-being, as defined by WHO, is a state where people realize their abilities, can cope with the normal stress of life, can work productively, and can contribute to their community [9]. Mental well-being is associated with many health mental health symptoms, including depression and health-related quality of life [10, 11]. Loneliness has also been identified to be a risk factor for depressive symptoms, while social support is known to be a protective factor mediating the effect of loneliness on depressive symptoms [12]. Previous studies showed that social support interventions (e.g., video conference programs) are effective to reduce loneliness and depressive symptoms [13, 14]. However, a study showed that family members' acceptance rate of videoconferencing use was low because of various reasons, such as preferring other alternatives (e.g., hiring a private caregiver) [15]. Visitation is still a preferred option by family and the older people living in LTCF, however, a systematic review showed that there are many reasons hampering family visitation of residents in LTCF, including family-residents’ relationships, employment, finances, and travel time [16]. Albeit the fact that family involvement is known to be associated with improved quality of life, evidence showed that family could only spend 4–9 h per week on the residents in LTCF and the involvement tend to decrease as the resident’s length of stay increases [17]. These interventions depending on family members do not benefit older residents without family members or having minimal family involvement.

Nature-based activities refer to the purposeful use of nature to formulate therapeutic activities which have been used to promote the mental well-being of older people (e.g., alleviating depressive symptoms, and promoting subjective happiness) [18–20]. However, nature-based activities commonly demand large outdoor spaces. Even indoor garden activities, the level of participation of older people living with physical disabilities is still expected to be limited. Reminiscence therapy refers to activities assisting older people to review their past, thus resulting in helping the resolution of the last development stage known as “ego integrity vs despair” in older people as derived from Erickson’s Psychosocial Development Theory [21]. A systematic review showed that reminiscence therapy can reduce depression in older people in LTCF [22]. Environmental stressors, mostly unmodifiable, are known to be associated with mental well-being in older people and outdoor leisure activities protectively mediate the effect of environmental stressors on the mental well-being of older people, particularly in LTCF [23]. An early study showed that several environmental factors in LTCFs cause.

depression in older people, including loss of independence, loss of freedom and continuity with their past life, loneliness, lack of privacy, and loss of autonomy due to the institutional regimen and regulations [24]. Evidence supported that outdoor recreational activities are protectively associated with depression in older people. However, their disability and the post-COVID-19 residual social distancing policy hinder them to participate in outdoor recreational activities.

Virtual reality (VR) is a computer system which can immerse users in a virtual environment by replacing the visual and aural environments to achieve a sense of presence so that users perceive themselves as being part of the virtual environment [25]. VR, therefore, renders LTCF possible to be environmentally unrestricted to providing LTCF residents with all protective elements promoting mental well-being. In the literature, as shown in a recent systematic review, VR has been used to promote mental health in older people mainly in three areas, including testing, training, and screening [26]. For training purposes, evidence supports that VR is effective to reduce loneliness and depressive symptoms in long-term care settings [27, 28]. One of the major strengths of using virtual encounters is that it does not involve family members and the variety of virtual experiences is not limited by space and environment. VR has also been used as a medium to deliver many therapeutic interventions (e.g., motor-cognitive training, gardening, outdoor leisure activities, tourism, and reminiscence) that are effective to promote health (e.g., cognitive function, physical function, mental well-being), feasible, and acceptable by older people, even in those who have various types of vulnerable conditions [29–33].

The use of VR in promoting the health of older people in LTCF is gaining popularity and its effects on the mental well-being of vulnerable groups, such as cognitive impairment, have been observed. However, most of the studies demonstrated the feasibility and preliminary effects in small feasibility or pilot studies only [30–33]. There is a lack of well-designed randomized controlled trials with an adequate number of samples to provide convincing evidence of its effects on mental well-being. Furthermore, some other interventions employed the element of physical activity (e.g., exergaming) in the intervention that they cannot be tolerated well by older people with physical disabilities [34]. Also, in the past, VR was a single-player activity, which may not induce peer social support through co-participation among participants in LTCF. Albeit the fact that VR has potential effects to promote the mental well-being of older people in LTCF, the effects of VR experience employing therapeutic elements without physical training components on social support and mental well-being are unproven in the context of older people with physical disabilities, in a randomized controlled trial with a representable number of sample.

### Objectives

This study aims to examine the effects of the therapeutic VR experience in older people with physical disabilities in LTCF in (1) increasing mental well-being, (2) reducing depressive symptoms, (3) reducing loneliness, (4) increasing health-related quality of life, and (5) increasing perceived social support.

### Trial design

This study employs a single-blinded, two-parallel-group (intervention-to-control group ratio = 1:1), non-inferiority, randomized controlled trial.

## Methods

The Standard Protocol Items: Recommendations for Interventional Trial (SPIRIT) 2013 guideline is followed to structure the report of this trial protocol and the details can be referred to Appendix: SPIRIT checklist [35].

### Study setting

This study will be conducted in the LTCF. Participants will be recruited from residential care homes for the elderly under the governance of the Social Welfare Department in Hong Kong [36]. The list of 171 residential care homes is shown in the Supplementary Document. Invitations will be extended to the residential care homes for the elderly and those who are interested will be invited to join the study. These residential care homes for the elderly provide residential care, meals, personal care, regular basic medical and nursing care, and social support for older people who suffer from poor health or physical/mental disabilities with a deficiency in activities of daily living but are mentally suitable for communal living [37].

### Eligibility criteria

#### Inclusion

1. Aged 60 years or above;
2. LTCF residents; and
3. Physical disabilities, as defined by the modified Barthel Index (MBI) score of ≤ 90 (i.e., moderately dependent or worse) [38].

#### Exclusion

1. Probably dementia, as defined by a Hong Kong version of the Montreal Cognitive Assessment (MoCA) score of < 19 [39];
2. Severe visual impairment, as defined by a lens-corrected visual acuity score of < 6/60 [40];
3. Severe hearing impairment, as defined by failed whispered voice test [41];
4. Bilateral upper limb paralysis, as defined by the Medical Research Council Muscle Power Scale of < 4 [42]; or
5. Participated in any VR activities in the past 6 months or concurrently.

### Intervention

#### Intervention key elements

As shown in Table 1, the intervention is described in nine key elements. The VR experience includes three therapeutic themes: natural scenery, outdoor leisure, and reminiscence because evidence showed that they are effective to promote mental health in older people [18, 22, 23]. In natural scenery, participants will be exposed to a virtual environment where they can visualize natural elements. In outdoor leisure, participants will be exposed to a virtual environment where they can visualize an outdoor relaxing environment. In reminiscence, participants will be exposed to a virtual environment where they can see old Hong Kong-themed environments.

**Table 1:** Intervention key elements

Intervention key elements	Contents
Components	• Natural scenery • Outdoor leisure • Reminiscence
Materials	• VR head-mount device • Tablet computer • Intervention implementation manual
Procedures	• Briefing • VR experience • Post-VR group discussion
Who	• Trained young volunteers
How	• Face-to-face
Where	• Bed or wheelchair in a shared room in LTCF
Duration	• One hour per session ◦ Briefing: 10 min ◦ VR experience: 20 min ◦ Post-VR group discussion: 30 min
Frequency	• 2 sessions per week
Course	• 6 weeks

The VR experience is launched on the participants using all-in-one VR head-mount devices. Tablet computers are used to optimize the settings by the interventionists. The image of each participant’s VR experience can also be mirrored on the tablet computers for the interventionists to monitor the progress of the intervention. A standardized intervention implementation manual depicts all the key steps in guiding the participants to explore the virtual environment.

In each session of the intervention, there are three parts: briefing, VR experience, and post-VR experience group discussion. In the briefing, VR head-mount devices are calibrated and put on the participants. Rules and control commands will also be introduced to participants. In the VR experience, interventionists will guide the participants to explore the virtual environment following a standardized intervention implementation manual. In the post-VR experience group discussion, the interventionists guide the participants to share their experiences (e.g., interesting encounters, difficulties, and associated anecdotes). The interventionists end the session by concluding their shared experiences.

The interventions can be implemented by the interventionists face-to-face with the participants in any room at the LTCF (e.g., activity room, participants’ bedrooms). Participants can join the intervention in a sitting position while they are in their beds or wheelchairs.

Each session lasts for approximately 1 h with 10 min spent on the briefing, 20 min spent on the VR experience, and 30 min spent on the post-VR group discussion. There are two sessions per week. The whole course lasts for 6 weeks and there are a total of 12 sessions.

#### Groups

Participants allocated to the intervention group will participate in the 6-week VR experience program. Participants allocated to the control will receive the usual care provided by the LTCF, such as personal care, regular basic medical and nursing care, and social support activities, as committed by the Social Welfare Department [37].

#### Criteria for discontinuing interventions

If actual or potential harms are identified, the trial steering team will report them to the advisory panel, which will decide to consider suspending or terminating the trials.

#### Interventionists

The interventionists are trained young volunteers who are studying in a post-secondary institution (i.e., Tung Wah College). Tung Wah College requires students to attend community services for 40 h as a form of service learning, which is also a graduation requirement. Serving as a volunteer interventionist in this project will be counted as community service required by the college. The same volunteer will deliver the intervention to the same group of participants throughout the 6-week intervention period. A train-the-trainer program will be provided by the research team to the young volunteers. The program will provide standardized training to the volunteer interventionist on skills of delivering the intervention and establishing rapport with the participants (e.g., communication skills). The research officer will supervise each training session to ensure that the interventions are delivered identically across groups. The interventionists deliver the intervention in an interventionist-to-participant ratio of 1:3. To ensure good intervention fidelity, the young volunteers will be qualified to become interventionists in this program after they have completed a 2-h train-the-trainer program. The contents of the train-the-trainer cover the activities for operating the VR experience system, familiarizing with the standardized intervention implementation manual, as well as the essential communication skills with older people with physical disabilities. The research team will pay site visits to the research venue and observe the intervention by checking the implementation steps against the implementation manual. Regular research team meetings will be conducted to solve problems hindering the interventionists from following the intervention protocol.

#### Intervention fidelity

Training will be provided for the interventionists, who are young volunteers, following an intervention protocol checklist that covers the key steps of the intervention. The attendance of the interventionists to provide the intervention will be recorded by the collaborating LCTF staff members. The trial steering team will randomly visit each site weekly to monitor the implementation of the intervention by the interventionists and check if the implementation adheres to the intervention protocol checklist. Regular research team meetings will be conducted to determine whether the interventionist has followed the intervention protocol. In case of deviation from the implementation is observed, remedial training for the interventionists will be delivered until the interventionist can return-demonstrate the intervention 100% correctly.

#### Relevant concomitant care

The participants allocated to the intervention group receive the same usual care provided by the LTCF. The research team does not interfere with the usual care provided to the participants in either group. However, participants are prohibited to participate in other additional activities related to VR experiences provided by other organizations during the intervention period.

### Outcomes

Demographic data including age, gender, level of education, number of chronic illnesses, length of stay in the LTCF, and experience of participation in VR activities will be collected. Demographic data will be collected at T0 (i.e., baseline, week 0) only. The primary outcome is mental well-being. The four secondary outcomes are depressive symptoms, loneliness, health-related quality of life, and perceived social support. All outcome data will be collected at T0 (i.e., baseline, week 0) and T1 (i.e., the week immediately after the completion of the intervention, week 7).

#### Mental well-being (primary outcome)

The World Health Organization Five Well-being Index (WHO-5) will be used to measure mental well-being [43]. WHO-5 comprises five items and each item is rated by a 6-point Likert scale from 0 (at no time) to 5 (all of the time). The total score ranges from 0 to 25. A higher score indicates better mental well-being. The Cantonese version of WHO-5 showed good internal consistency (*α* = 0.86) and good concurrent validity with quality of life (*r*= 0.41–0.51) [43].

#### Depressive symptoms

The 9-item Patient Health Questionnaire (PHQ-9) will be used to measure depressive symptoms over the past 2 weeks [44]. PHQ-9 comprises nine items and each item is rated by a 4-point Likert scale from 0 (not at all) to 3 (nearly every day). The total score ranges from 0 to 27. A higher score indicates greater severity of depressive symptoms. The Cantonese version of PHQ-9 showed good internal consistency (*α* = 0.82), test–retest reliability (*r* = 0.76), and satisfactory concurrent validity with the mental component of quality of life (*r*=  − 0.60) [44].

#### Loneliness

The Chinese version of the 6-item De Jong Gierveld Loneliness Scale (DJGLS) will be used to measure loneliness [45]. The scale comprises three items measuring emotional loneliness and the other three items on social loneliness. Each item is rated on a 3-point scale (0 = no, 1 = more or less, 1 = yes). The total score ranges from 0 to 6. A higher score indicates a greater level of loneliness. DJGLS is reliable with good internal consistency (Cronbach’s = 0.76) and excellent inter-rater reliability (ICC = 0.98–1.00) and valid with good correlation with the answers to the direct question of loneliness (*r*= 0.71) [45].

#### Health-related quality of life

The Hong Kong version of the EuroQol 5-dimensions instrument with a five-level scale (EQ-5D-5L) will be used to measure health-related quality of life [46]. EQ-5D-5L consists of two parts: descriptive system and visual analog scale. The descriptive system has five dimensions: mobility, self-care, usual activities, pain/discomfort, and anxiety/depression. Each dimension is rated on a five-level scale (no problem, slight problem, moderate problems, severe problems, and extreme problems). All five health states defined by EQ-eD-5L can be converted to an index score by using a utility based on the Hong Kong value set. The utility value ranges from − 1 to 1 (0 indicates death, < 0 indicates worse than death, and 1 indicates full health). A systematic review showed that its test–retest reliability is excellent (ICC = 0.7–1.0) and its convergent validity is good (pooled rho = 0.756 for multi-attribute utility instruments) [47].

#### Perceived social support

The Chinese version of the Multiple Scale of Perceived Social Support (MSPSS) will be used to measure perceived social support [48]. MSPSS comprises 12 items from three subscales: family, friends, and significant others. Each item is rated on a 7-point Likert scale ranging from 1 (very strongly disagree) to 7 (very strongly agree). A total score is the summation of the results of all items. The possible score ranges from 12 to 84, with a higher score indicating a higher level of perceived social support. MSPSS has good internal consistency (Cronbach’s *α* = 0.92), good test–retest reliability (ICC = 0.65), and good convergent validity (*r*≥ 0.80) [48].

### Participant timeline

**Fig. 1:**
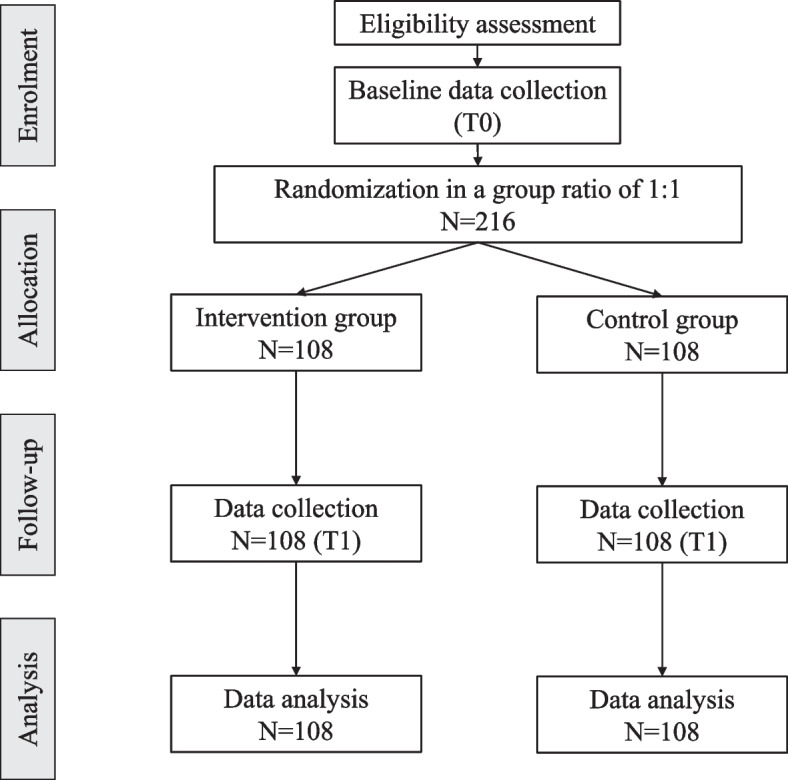
CONSORT flow diagram: T0: Baseline for pre-treatment assessment (i.e., week 0), T1: The week immediately after the intervention (i.e., week 7)

As shown in Table 2 and Fig. 1, potential participants will be recruited in the phase of enrolment, in which the eligibility screen and informed consent will be implemented. Then, in the phase of pre-treatment assessment (i.e., T0, baseline, week 0), demographic and outcome data will be collected. Subsequently, in the phase of allocation, participants will be randomly allocated to either the intervention group or the control group. Then, interventions will be implemented. In the phase of post-treatment assessment (i.e., T1, the week immediately after the completion of the intervention, week 7), outcome data will be collected once again.

**Table 2:** Schedule of enrolment, intervention, and assessments (SPIRIT figure)

Timepoint	Enrolment	Pre-treatment assessment	Allocation	Intervention	Post-treatment assessment
		T0			T1
		Week 0			Week 7
Enrolment					
Eligibility screen	x				
Informed consent	x				
Group allocation			x		
Interventions					
Therapeutic VR experience				x	
Control				x	
Data collection					
Demographic		x			
Outcome					
Mental well-being		x			x
Depressive symptoms		x			x
Loneliness		x			x
Health-related QoL		x			x
Perceived social support		x			x

### Sample size

In the literature, there is a lack of randomized controlled trials evaluating the effect of therapeutic VR experience on mental well-being using specifically in older people with physical disabilities. A similar study evaluating the effects of feedback-based virtual reality exercise on mental health (measured by the mental component summary of the Short-Form Health Survey–36 items) in older women showed that the between-group effect size at the follow-up timepoint is moderate (i.e., *d*= 0.407) [49].

A priori power analysis using G*Power employing the statistical test of two-tailed *t*-test (means: difference between two independent means of two groups) is employed. We assume that The level of significance is set at 0.05. The effect size *d*is set at 0.407 [49]. The power is set at 0.8. The group allocation ratio is set at 1:1. It is estimated that 192 samples are needed with 96 samples in each group. Considering the attrition rate of 11.3% referring to another similar study examining the effects of a VR hands-on horticultural therapy on institutionalized older people [28], the total sample size of the proposed study is estimated to be 216.

### Recruitment

Health talks on the topic of the use of VR for promoting mental health will be delivered in the collaborating LTCF. The residents, their family caregivers, and the staff members will be invited to attend. After the talks, recruitment will immediately begin at the scene. Both electronic (e-poster, short videos) and printed (e.g., flyers) promotional materials will be produced. The materials will be sent to the collaborating LTCF before the health talks. The collaborating LTCF will be invited to share the materials with potential participants and their family members via post, email, WhatsApp, and social media (e.g., Facebook). The project team and the staff members of the collaborating LTCF will be responsible for the recruitment continuously until the potential participants are exhausted. To ensure a high recruitment rate, the eligibility criteria and research information materials will be provided and explained to staff members of the collaborating LTCF in a research briefing session hosted by the research team. This is to ensure that the participants invited by the LTCF staff members are more likely to be eligible to join this study. It is estimated that the recruitment rate will be above 50%.

### Assignment of interventions

#### Allocation

Permuted block randomization will be used to randomly select block sizes and a group ratio of 1:1 will be used [50]. Participants will be randomly allocated into groups individually following the order of the randomized group sequence. The random allocation sequence list will be generated by the web-based application Research Randomizer [51]. The list will be generated by an independent research team member, who will not participate in any other parts of the proposed study. The research team member will assign group labels to the participants based on their sequence of entries to the study referring to the random group allocation sequence list. As a result, the group allocation sequence is concealed from other research team members.

#### Blinding

In the proposed study, only the outcome assessor will be blinded to the group labels. Participants and interventionists are not possible to be blinded in this study. The group labels will not be known to the outcome assessors and will not appear on any documents that the outcome assessors can access. The participants, family members, and staff members of the LTCF are prohibited to disclose the group labels of the participants to the outcome assessors.

### Data collection and management

Data will be collected by the project implementation team, including one research officer and one research assistant, as well as a group of trained student helpers who are blinded to the group label for data collection in the phase of post-treatment assessment. Data will be collected using a computerized online data collection application Qualtrics (www.qualtrics.com). Qualtrics can prompt the data collectors for the unfilled data field and the data collector must provide reasons for leaving the data field blanks before they can submit the data, to minimize the risk of unintentional missing data. On the day immediately after the data collection, the data collectors will conduct preliminary data analysis to do the range check to ensure that the data are complete and the data are entered correctly. When there are missing data or out-of-range data noted, the project implementation team will investigate the causes and re-conduct the data collection as immediately as possible. Qualtrics employs many safety methods to ensure the confidentiality of the data on the server (e.g., high-end firewall system, encryption) [52]. The data on Qualitrics will be saved on the password-protected cloud server at Tung Wah College for at least seven years according to the requirement of the ethics committee after each round of data collection. Only the research team members have access right to the final full dataset after the completion of the study. To promote participant retention, a completion certificate issued by Tung Wah College will be presented to participants who completed the intervention and follow-up data collection in a celebratory ceremony. For participants who discontinue the intervention or deviate from the intervention protocol, data on all five outcomes (i.e., mental well-being, depressive symptoms, loneliness, health-related quality of life, and perceived social support) will also be collected in the week after the normal completion of the intervention (i.e., week 7).

### Statistical methods

Demographic and outcome data collected at baseline will be reported either as means with standard deviation or as frequencies with percentages according to their levels of measurement as a whole sample and by groups. Generalized estimating equations (GEE) will be employed to separately test the hypothesis on the five outcomes as dependent variables (i.e., mental well-being, depressive symptoms, loneliness, health-related quality of life, and perceived social support), The independent variables will be the same across all GEEs: the group (two categories: intervention and control groups), the timepoint (two categories: T0 and T1), and the interaction term (i.e., group × timepoint). The primary interpretation of the results will be based on the intention-to-treat analysis without adjusting for covariates [53, 54]. Sensitivity analysis will be conducted to compare the results of different analysis methods, including intention-to-treat, as-treated, and adherers-only analyses [55]. The level of significance will be set at 0.05. Missing data (e.g., due to dropout or death) will be managed following Jakobsen’s algorithm with multiple methods (e.g., multiple imputation) according to the missing pattern [56].

### Monitoring

A trial steering team includes one academic in the disciplines of nursing (i.e., the author RK) and a social worker (i.e., the author OS). OS is the in-charge of the elderly service of a non-government organization and will direct the whole study process. The trial steering team coordinates the study venues, budget, and study progress. The project implementation team comprising one research officer and a team of nursing student helpers will be responsible to implement the research procedures (e.g., eligibility screening, informed consent, and data collection). An advisory panel comprising one academic in psychiatry (i.e., the author LL) and social entrepreneur and social service director (i.e., the author RY) will provide advice to the project and monitor the quality of the implementation (e.g., data collection, intervention implementation, data analysis). The data management committee comprising two academics in the disciplines of nursing (i.e., the author SC) and occupation therapy (i.e., the author FN) monitors the quality of data collection and data analysis. The trial steering team will meet the project implementation team bi-weekly to monitor the adherence of the implementation to the research protocol and provide suggestions to solve the operational problems faced by the project implementation team. The advisory panel and data monitoring committee are independent of the trial sponsor (i.e., the author RK) and competing interests. The research team, including the trial steering committee and the data monitoring committee, report to and seek advice from the advisory panel at a 3-month interval for higher-level issues, including suspicion of adverse effects, suspension of the trial, and modification of the research protocol.

Adverse effects commonly reported on older people after using virtual reality-based interventions include cybersickness, fall injuries, dizziness, and eyestrain, although the incidence is low [57]. To ensure safety, all participants will attend the intervention in a sitting position to minimize the risk of falls. To minimize the risk of cybersickness, dizziness, and eyestrain, the VR experience in each section is limited to 20 min only. After the completion of the intervention, the interventionist will stay with the participants for 15 min more and examine the participants for any common adverse effects or discomforts. Basic training will also be provided to the interventionists to identify common adverse effects (e.g., signs of cybersickness) and provide immediate management for mild symptoms (e.g., lying the participants flat on the bed with eyes closed for a few minutes to prevent fall injuries). The LTCF staff members will be trained to observe common adverse effects on the participants. All suspected adverse effects will be recorded in a logbook. The interventionists and LTCF staff members will inform the trial steering committee when suspected adverse effects are observed. If the adverse effects are severe (e.g., fall injuries), participants will be immediately sent to a hospital for immediate treatment. If the adverse effect is severe, the case of suspected adverse effects will also be discussed in the advisory panel to determine whether it is related to the intervention. The project implementation team will meet the participants and the staff members of the collaborative LTCF regularly. If suspected adverse events related to the interventions are reported by the participants, interventionists, or LTCF staff members, the implementation team will report them to the trial steering team. Judged by the trial steering team, if the events are likely to be related to the intervention, the advisory panel will be informed and the panel will decide if the adverse events are related to the trial. Subsequently, the advisory panel will decide to either continue the trial, withdraw the affected participants from the trial, revise the trial protocol, or terminate the trial. An independent audit company will be hired to audit the trial and provide an audit report once per year.

### Ethics and dissemination

This study has been approved by the Research Ethics Committee of Tung Wah College, Hong Kong (reference number: REC2023158).

In case there are changes in the protocol, the research team will notify the sponsor, funder, and Research Ethics Committee. Then, the research venues (i.e., centers for older people) will be informed and a copy of the revised protocol will be sent to them. If there are any deviations from the protocol, the changes will be fully documented using a breach report form and the details in the clinical trial registry will also be updated.

All participants will be asked to give their written informed consent to participate in the proposed study. The written consent will be obtained by a research assistant after explaining the study to the participants guided by a standardized information sheet. No identifying images or other personal or clinical details of participants are presented here or will be presented in reports of the trial results.

Details of the protocol, participant-level data, statistical code, the written consent form, and the information sheet about the study to be provided to participants are available from the corresponding author upon request. After the completion of the study, only the research team members have the right to access the final full dataset. Given that there are no anticipated serious harms, there are no provisions for ancillary and post-trial care for compensation to those who suffer harm from trial participation.

The findings will be disseminated in peer-reviewed journals, presented at international and local conferences with related themes (e.g., mental health, gerontology, therapeutic virtual reality, and LTCF), and shared in local media (e.g., e-newspaper).

## Discussion

The mental well-being of older LTCF residents living with physical disabilities is a significant problem, and there are effective therapeutic elements available (e.g., family visitation, reminiscence, nature-based activities, outdoor activities) [18, 19, 22, 23, 58]; however, their physical disabilities may impair their exposure to these activities and the environmental stressors may hinder the beneficial effects to be yielded [3, 23]. Virtual reality-based activities delivered by young volunteers allow these effective therapeutic elements to be exposed to the older LTCF residents living with physical disabilities because they can be implemented on the bed or wheelchair of the participants. The use of information and communication technology products (e.g., VR, smartphone, wearable devices) in older people for health promotion is highly feasible and acceptable by older people, but they are mostly used in community-dwelling older people [29, 59–62]. If the intervention proves effective in promoting mental well-being, older LTCF residents living with physical disabilities are expected to have a better quality of life. After the completion of the project, the developed VR program and the implementation manual will be uploaded to a platform which allows LTCF staff members who did not participate in this study to freely download and use the program to promote the mental well-being of older LTCF residents living with disabilities.

## Trial status

The trial is currently in the phase of VR software development. Recruitment is expected to begin on 15 Feb 2024 and until 14 February 2025.

### Supplementary Information

**Additional file 1. **List of subvented, self-financing and contract residential care homes for the elderly providing subsidised places for the elderly (As at 30.6.2023)

**Additional file 2.**

## Data Availability

The data will be provided upon request.

## Abbreviations

LTCF: Long-term care facilities
COVID-19: 2019 Novel coronavirus
VR: Virtual reality
SPIRIT: Standard Protocol Items: Recommendations for Interventional Trial
MBI: Modified Barthel Index
MoCA: Montreal Cognitive Assessment
WHO-5: World Health Organization Five Well-being Index
PHQ-9: 9-Item Patient Health Questionnaire
DJGLS: De Jong Gierveld Loneliness Scale
EQ-5D-5L: EuroQol 5-dimensions instrument with a five-level scale
MSPSS: Multiple Scale of Perceived Social Support
ICC: Intraclass correlation coefficient
GEE: Generalized estimating equations
