# Supplementary material for: The effects of therapeutic virtual reality experience to promote mental well-being in older people living with physical disabilities in long-term care facilities
Source: Trials. 2023 Aug 26;24:558. doi: 10.1186/s13063-023-07592-7 (PMC10464193; doi:10.1186/s13063-023-07592-7)
Supplement: Supplementary file 1 — Additional file 1. List of subvented, self-financing and contract residential care homes for the elderly providing subsidised places for the elderly (As at 30.6.2023) [file 13063_2023_7592_MOESM1_ESM.pdf]

List of Subvented, Self-financing and Contract Residential Care Homes for the Elderly Providing Subsidised Places for the Elderly (As at 30.6.2023)
提供資助安老服務宿位的津助院舍、自負盈虧院舍及合約院舍名單 (截至 30.6.2023)

S/N 編號	District 地區	Agency 機構	Name of Home 院舍名稱	Address 地址	Tel. 電話	Fax 傳真	Type of Service 服務類別	Type of Place 宿位種類					Total 總數	Sex 性別	Religion 宗教	Diet 膳食
								**Hostel for the Elderly** 長者宿舍	**Home for the Aged** 安老院	**Care-and-Attention** 護理安老院	**Care-and-Attention Providing a Continuum-of-Care** 提供持續照顧的護理安老院	**Nursing Home** 護養院				
1	Eastern 東區	Tung Wah Group of Hospitals 東華三院	TWGHs Lee See Ping Home for the Elderly 東華三院馬李示聘安老院†	Units 405 to 432, Chin Hing House, Hing Wah (II) Estate, Chai Wan, Hong Kong 香港柴灣興華（二）邨展興樓405至432室	25567952	25581136	Conversion Home Providing COC	0	0	0	55	0	**55**	M/F 男/女	Nil 無	Non-vegetarian 非素食
2	Eastern 東區	Tung Wah Group of Hospitals 東華三院	TWGHs Fong Shu Chuen Care and Attention Home 東華三院方樹泉護理安老院@	2/F to 5/F, Fong Shu Chuen Social Service Building, 6 Po Man Street, Shau Kei Wan, Hong Kong 香港筲箕灣寶文街6號方樹泉社會服務大樓2字樓至5字樓	29670991	29154955	C&A Home Providing COC	0	0	0	174	0	**174**	M/F 男/女	Nil 無	Non-vegetarian 非素食
3	Eastern 東區	Tung Wah Group of Hospitals 東華三院	TWGHs Women's Welfare Club Western District, Hong Kong Residential Care Home for the Elderly 東華三院香港西區婦女福利會護養安老院*	No. 501, 5/F, Oi Sin House, Oi Tung Estate, Shau Kei Wan, Hong Kong 香港筲箕灣愛東邨愛善樓5字樓501室	31562111	31561456	Contract Home	0	0	0	10	90	**100**	M/F 男/女	Nil 無	Non-vegetarian 非素食
4	Eastern 東區	Women's Welfare Club (Eastern District) Hong Kong 香港東區婦女福利會	Women's Welfare Club (Eastern District) Hong Kong Ng Siu Mui Home cum Care and Attention Unit for the Elderly 香港東區婦女福利會伍少梅安老護理院†@	7/F, Car Park Block II, Yiu Tung Estate, Shau Kei Wan, Hong Kong 香港筲箕灣耀東邨2號停車場7字樓	29674888	25600276	Conversion Home Providing COC	0	0	0	101	0	**101**	M/F 男/女	Nil 無	Non-vegetarian 非素食
5	Eastern 東區	The Lutheran Church-Hong Kong Synod Limited 香港路德會有限公司	Hong Kong Lutheran Social Service Mr. & Mrs. Lawrence Wong Lutheran Home for the Elderly 香港路德會社會服務處路德會黃鎮林伉儷安老院†@	G/F & 1/F, Sui Ming House & Sui Tai House, Siu Sai Wan Estate, Chai Wan, Hong Kong 香港柴灣小西灣邨瑞明樓及瑞泰樓地下及1樓	25053683	28978598	Conversion Home Providing COC	0	0	0	118	0	**118**	M/F 男/女	Christian 基督教	Non-vegetarian 非素食
6	Eastern 東區	St James' Settlement 聖雅各福群會	Scenic Resort (Nursing Home) 雅明灣畔護養院*	G/F (part) and 1/F - 4/F, Oi Po House, Oi Tung Estate, Shau Kei Wan, Hong Kong 香港筲箕灣愛東邨愛寶樓地下(部分)及一字樓至四字樓	31954215	31954205	Contract Home	0	0	0	8	72	**80**	M/F 男/女	Christian 基督教	Non-vegetarian 非素食
7	Wan Chai 灣仔區	Heung Hoi Ching Kok Lin Association 香海正覺蓮社	Heung Hoi Ching Kok Lin Association Buddhist Li Ka Shing Care and Attention Home for the Elderly 香海正覺蓮社主辦佛教李嘉誠護理安老院@	G/F to 8/F and 10/F (H.K.I.L. 8721), 133 Tai Hang Road, Hong Kong 香港第8721內地段大坑道133號地下至8字樓及10字樓	28811801	25771831	C&A Home Providing COC	0	0	0	262	0	**262**	M/F 男/女	Buddhist 佛教	Vegetarian/ Non-vegetarian 素食/ 非素食
8	Wan Chai 灣仔區	Hong Kong Tuberculosis, Chest and Heart Diseases Association 香港防癆心臟及胸病協會	Freni Care and Attention Home 傅麗儀護理安老院@	1H, Shiu Fai Terrace, Wan Chai, Hong Kong 香港灣仔肇輝臺1號H	22392388	25919223	C&A Home Providing COC	0	0	0	200	0	**200**	M/F 男/女	Nil 無	Non-vegetarian 非素食
9	Wan Chai 灣仔區	Po Leung Kuk 保良局	Po Leung Kuk Wan Chai Home for the Elderly cum Day Care Centre for the Elderly 保良局灣仔護老院暨長者日間護理中心*	Portions of Level 1.2 (below ground), G/F, M/F, 1/F and 5/F, 2/F and 3/F, 7A Cross Street, Wan Chai, Hong Kong 香港灣仔交加街7 號A水平1.2 層（部分）、地下（部分）、閣樓（部分）、一樓（部分）、二樓、三樓及五樓（部分）	26181130	26181737	Contract Home	0	0	0	6	54	**60**	M/F 男/女	Nil 無	Non-vegetarian 非素食
10	Central/ Western 中西區	Tung Wah Group of Hospitals 東華三院	TWGHs Hui Mok Tak Yu Care and Attention Home 東華三院許莫德瑜護理安老院@	1/F to 4/F & part of 5/F, Tower 125, 40 to 48 Tai Ping Shan Street, 7 to 15 Po Yan Street, and 3 Po Yee Street, Sheung Wan, Hong Kong 香港上環太平山街40至48號普仁街7至15號及普義街3號世銀花苑1字樓至4字樓及5字樓部分	28030099	29151177	C&A Home Providing COC	0	0	0	153	0	**153**	M/F 男/女	Nil 無	Non-vegetarian 非素食
11	Central/ Western 中西區	Po Leung Kuk 保良局	Po Leung Kuk Chan Au Big Yan Home for the Elderly 保良局陳區碧茵頤養院†@	12 Belcher's Street, Kennedy Town, Hong Kong 香港堅尼地城卑路乍街12號	28171858	28183174	Conversion Home Providing COC	0	0	0	83	0	**83**	M/F 男/女	Nil 無	Non-vegetarian 非素食

List of Subvented, Self-financing and Contract Residential Care Homes for the Elderly Providing Subsidised Places for the Elderly (As at 30.6.2023)
提供資助安老服務宿位的津助院舍、自負盈虧院舍及合約院舍名單 (截至 30.6.2023)

S/N<br>編號	District<br>地區	Agency<br>機構	Name of Home<br>院舍名稱	Address<br>地址	Tel.<br>電話	Fax<br>傳真	Type of Service<br>服務類別	Type of Place<br>宿位種類					Total<br>總數	Sex<br>性別	Religion<br>宗教	Diet<br>膳食
								**Hostel for the Elderly**<br>長者宿舍	**Home for the Aged**<br>安老院	**Care-and-Attention**<br>護理安老院	**Care-and-Attention Providing a Continuum-of-Care**<br>提供持續照顧的護理安老院	**Nursing Home**<br>護養院				
12	Central/ Western<br>中西區	Caritas - Hong Kong<br>香港明愛	Caritas Evergreen Home<br>明愛恩翠苑*	LG/F (portion), 1/F and 2/F, Sai Ying Pun Community Complex, 2 High Street, Sai Ying Pun, Hong Kong<br>香港西營盤高街2號西營盤社區綜合大樓低層（部分）、1字樓及2字樓	25596685	25596072	Contract Home	0	0	0	12	109	**121**	M/F<br>男/女	Catholic<br>天主教	Non-vegetarian<br>非素食
13	Central/ Western<br>中西區	Po Leung Kuk<br>保良局	Po Leung Kuk Sai Ying Pun Home for the Elderly cum Day Care Centre for the Elderly<br>保良局西營盤護老院暨長者日間護理中心*	Parts of G/F, 1/F, 2/F, 3/F and 5/F, 8 First Street, Sai Ying Pun, Hong Kong<br>香港西營盤第一街8號地下、1樓、2樓、3樓及5樓（各樓層部分）	2796 3166	2796 3177	Contract Home	0	0	0	6	51	**57**	M/F<br>男/女	Nil<br>無	Non-vegetarian<br>非素食
14	Central/ Western<br>中西區	Po Leung Kuk<br>保良局	Po Leung Kuk Kwok Law Kwai Chun Home for the Elderly<br>保良局郭羅桂珍護老院*	G/F to 4/F and portion of roof, 28 Ko Shing Street, Sheung Wan, Hong Kong<br>香港上環高陞街28號地下至4字樓及部分天台	28586233	28587133	Contract Home	0	0	0	3	31	**34**	M/F<br>男/女	Nil<br>無	Non-vegetarian<br>非素食
15	Islands<br>離島	Caritas - Hong Kong<br>香港明愛	Caritas Fu Tung Home<br>明愛富東苑	G/F, 1/F & 2/F, Tung Shing House, Fu Tung Estate, Tung Chung, Lantau Island<br>大嶼山東涌富東邨東盛樓地下、1字樓及2字樓	21093302	21093588	C&A Home Providing COC	0	0	0	110	0	**110**	M/F<br>男/女	Catholic<br>天主教	Non-vegetarian<br>非素食
16	Islands<br>離島	Chung Shak Hei (Cheung Chau) Home for the Aged Limited<br>鍾錫熙長洲安老院有限公司	Chung Shak Hei (Cheung Chau) Home for the Aged<br>鍾錫熙長洲安老院@	Pak She, Cheung Chau<br>長洲北社	29810002	29815220	Combined Home	0	9	71	0	0	**80**	M/F<br>男/女	Nil<br>無	Non-vegetarian<br>非素食
17	Islands<br>離島	Chung Shak Hei (Cheung Chau) Home for the Aged Limited<br>鍾錫熙長洲安老院有限公司	Wan Ho Kan Care and Attention Home<br>溫浩根護理安老院@	9 Wo Shun Lane, Pak She, Cheung Chau<br>長洲北社和順里9號	29810820	29869083	C&A Home Providing COC	0	0	0	112	0	**112**	M/F<br>男/女	Nil<br>無	Non-vegetarian<br>非素食
18	Islands<br>離島	Po Leung Kuk<br>保良局	Po Leung Kuk Tung Chung Home for the Elderly<br>保良局東涌護老院*	Portions of G/F to 2/F and 3/F to 5/F, Tung Chung Municipal Services Building, 39 Man Tung Road, Tung Chung, New Territories<br>新界東涌文東路39號東涌市政大樓地下至2字樓部分及3字樓至5字樓	21092038	21092032	Contract Home	0	0	0	8	67	**75**	M/F<br>男/女	Nil<br>無	Non-vegetarian<br>非素食
19	Islands<br>離島	World Castle Limited<br>偉其有限公司	Tung Chung Silverjoy<br>耆樂東涌*	G/F (portion), 1/F (portion) and 2/F (portion), Ying Fook House, Ying Tung Estate, 12 Ying Tung Road, Tung Chung, New Territories<br>新界東涌迎東路12號迎東邨迎福樓地下（部分）、一樓（部分）及二樓（部分）	28898822	28898200	Contract Home	0	0	0	7	63	**70**	M/F<br>男/女	Nil<br>無	Non-vegetarian<br>非素食
20	Southern<br>南區	Chuk Lam Ming Tong Limited<br>竹林明堂有限公司	Chuk Lam Ming Tong Care & Attention Home for the Aged<br>竹林明堂護理安老院†@	5 Sha Wan Drive, Pok Fu Lam, Hong Kong<br>香港薄扶林沙灣徑5號	28172281	28188566	Conversion Home Providing COC	0	0	0	175	0	**175**	M/F<br>男/女	Buddhist/ Taoist/ Confucian<br>佛/道/孔教	Vegetarian/ Non-vegetarian<br>素食/<br>非素食
21	Southern<br>南區	Tung Wah Group of Hospitals<br>東華三院	TWGHs Jockey Club Harmony Villa<br>東華三院賽馬會朗逸居†@	LG/F (portion), G/F (portion), M/F, 1/F and 2/F, Jockey Club Sunshine Complex for the Elderly, 29 Nam Long Shan Road, Wong Chuk Hang, Hong Kong<br>香港黃竹坑南朗山道29號東華三院賽馬會松朗安老綜合中心地下低層部分、地下部分、閣樓、1樓及2樓	22923488	22923400	Conversion Home Providing COC	0	0	0	167	0	**167**	M/F<br>男/女	Nil<br>無	Non-vegetarian<br>非素食

List of Subvented, Self-financing and Contract Residential Care Homes for the Elderly Providing Subsidised Places for the Elderly (As at 30.6.2023)
提供資助安老服務宿位的津助院舍、自負盈虧院舍及合約院舍名單 (截至 30.6.2023)

S/N 編號	District 地區	Agency 機構	Name of Home 院舍名稱	Address 地址	Tel. 電話	Fax 傳真	Type of Service 服務類別	Type of Place 宿位種類<br>Hostel for the Elderly 長者宿舍	Home for the Aged 安老院	Care-and-Attention 護理安老院	Care-and-Attention Providing a Continuum-of-Care 提供持續照顧的護理安老院	Nursing Home 護養院	Total 總數	Sex 性別	Religion 宗教	Diet 膳食
22	Southern 南區	Tung Wah Group of Hospitals 東華三院	TWGHs Yu Chun Keung Memorial Care and Attention Home 東華三院余振強紀念護理安老院@	Portion of Lower G/F, and G/F, East and West Wings of 1/F and 2/F and West Wing of 3/F, Block B, Wong Chuk Hang Complex for the Elderly, 2 Wong Chuk Hang Path, Wong Chuk Hang, Hong Kong 香港黃竹坑黃竹坑徑2號黃竹坑老人服務綜合大樓B座地下及地下低層部分、1字樓及2字樓東西翼及3字樓西翼	28142866	25183801	C&A Home Providing COC	0	0	0	200	0	**200**	M/F 男/女	Nil 無	Non-vegetarian 非素食
23	Southern 南區	Po Leung Kuk 保良局	Po Leung Kuk Wong Chuk Hang Service for the Elderly - 1984 Care and Attention Home cum Madam Aw Tan Kyi Kyi Home for the Aged 保良局黃竹坑護理安老中心 - 甲子護理安老院暨胡陳金枝安老院†@	A.I.L. 413, Nam Fung Road (also known as No. 1 Wong Chuk Hang Path), Wong Chuk Hang, Hong Kong) 香港黃竹坑南豐路A.I.L.413（亦稱黃竹坑徑1號）	25540020	25187506	Conversion Home Providing COC	0	0	0	117	0	**117**	M/F 男/女	Nil 無	Non-vegetarian 非素食
24	Southern 南區	Hong Kong Society for the Aged (The) 香港耆康老人福利會	SAGE Bradbury Home for the Elderly 耆康會白普理護理安老院@	62 Tin Wan Street, Aberdeen, Hong Kong 香港香港仔田灣街62號	28701010	25808777	C&A Home Providing COC	0	0	0	196	0	**196**	M/F 男/女	Nil 無	Non-vegetarian 非素食
25	Southern 南區	Hong Kong Society for the Aged (The) 香港耆康老人福利會	SAGE Quan Chuen Home for the Elderly 耆康會關泉護理安老院@	60 Tin Wan Street, Aberdeen, Hong Kong. 香港香港仔田灣街60號	28701010	25808777	C&A Home Providing COC	0	0	0	196	0	**196**	M/F 男/女	Nil 無	Non-vegetarian 非素食
26	Southern 南區	Hong Kong Society for the Aged (The) 香港耆康老人福利會	SAGE Madam Ho Sin Hang Home for the Elderly 耆康會何善衡夫人敬老院@	1 Sha Wan Drive, Pok Fu Lam, Hong Kong 香港薄扶林沙灣徑1號	22801300	28166139	C&A Home Providing COC	0	0	0	190	0	**190**	M/F 男/女	Nil 無	Non-vegetarian 非素食
27	Southern 南區	Hong Kong Young Women's Christian Association 香港基督教女青年會	Hong Kong Young Women's Christian Association Cheng Pon Hing Care and Attention Home for the Elderly 香港基督教女青年會鄭傍卿護理安老苑†	Unit nos. 101 to 108, 117 to 124 and 201 to 224, Tung Yip House, Lei Tung Estate, Ap Lei Chau, Hong Kong 香港鴨脷洲利東邨東業樓101至108室、117至124室及201至224室	28743663	28742236	Conversion Home Providing COC	0	0	0	108	0	**108**	M/F 男/女	Christian 基督教	Non-vegetarian 非素食
28	Southern 南區	Hong Kong Christian Service 香港基督教服務處	Hong Kong Christian Service - Wah Hong Home for the Elderly 香港基督教服務處華康安老院†	Unit 217-234 & 317-334, Wah Hong House, Wah Fu Estate, Aberdeen, Hong Kong 香港香港仔華富邨華康樓217-234室及317-334室	25510980	25381737	Conversion Home Providing COC	0	0	0	40	0	**40**	M/F 男/女	Christian 基督教	Non-vegetarian 非素食
29	Southern 南區	Hong Kong & Macau Lutheran Church Social Service Limited 港澳信義會社會服務有限公司	Hong Kong & Macau Lutheran Church Wai Kei Hostel 港澳信義會懷耆苑†@	Room 201 to 232, 2/F, Lei Ning House, Ap Lei Chau Estate, Hong Kong 香港鴨脷洲邨利寧樓2字樓201至232室	25530181	28731812	Conversion Home Providing COC	0	0	0	76	0	**76**	M/F 男/女	Christian 基督教	Non-vegetarian 非素食
30	Shamshuipo 深水埗區	Tung Wah Group of Hospitals 東華三院	TWGHs Ma Cheng Shuk Ying Home for the Elderly 東華三院馬鄭淑英安老院†	Wing C, G/F and 2/F, Wo Muk House, Lei Cheng Uk Estate, Sham Shui Po, Kowloon 九龍深水埗李鄭屋邨和睦樓地下翼及2樓C翼	27285226	27251771	Conversion Home Providing COC	0	0	0	75	0	**75**	M/F 男/女	Nil 無	Non-vegetarian 非素食
31	Shamshuipo 深水埗區	Po Leung Kuk 保良局	Po Leung Kuk Eco-Home for the Senior cum Sunny Green Day Care Centre for the Senior 保良局癸未年樂頤居暨耆安長者日間護理中心*	Podium Level (part) & Level 2, Fu Yun House, Fu Cheong Estate, Sham Shui Po, Kowloon 九龍深水埗富昌邨富潤樓平台（部分）及二樓	22674579	22674250	Contract Home	0	0	0	7	64	**71**	M/F 男/女	Nil 無	Non-vegetarian 非素食
32	Shamshuipo 深水埗區	Caritas - Hong Kong 香港明愛	Caritas Lai Kok Home 明愛麗閣苑†@	Units 201 to 227, Lai Lo House, Lai Kok Estate, Sham Shui Po, Kowloon 九龍深水埗麗閣邨麗蘿樓201至227室	27289217	29580962	Conversion Home Providing COC	0	0	0	80	0	**80**	M/F 男/女	Catholic 天主教	Non-vegetarian 非素食
33	Shamshuipo 深水埗區	Hong Kong Young Women's Christian Association 香港基督教女青年會	Hong Kong Young Women's Christian Association Wan Wah Care and Attention Home for the Elderly 香港基督教女青年會雲華護理安老苑@	Wing B and C of G/F, and Wing A to D of 1/F, Lai Lim House and Lai Wing House, Lai On Estate, Sham Shui Po, Kowloon 九龍深水埗麗安邨麗廉樓及麗榮樓地下B、C翼及1字樓A至D翼	27083677	27291359	C&A Home Providing COC	0	0	0	151	0	**151**	M/F 男/女	Christian 基督教	Non-vegetarian 非素食

List of Subvented, Self-financing and Contract Residential Care Homes for the Elderly Providing Subsidised Places for the Elderly (As at 30.6.2023)
提供資助安老服務宿位的津助院舍、自負盈虧院舍及合約院舍名單 (截至 30.6.2023)

S/N 編號	District 地區	Agency 機構	Name of Home 院舍名稱	Address 地址	Tel. 電話	Fax 傳真	Type of Service 服務類別	Type of Place 宿位種類					Total 總數	Sex 性別	Religion 宗教	Diet 膳食
								Hostel for the Elderly 長者宿舍	Home for the Aged 安老院	Care-and-Attention 護理安老院	Care-and-Attention Providing a Continuum-of-Care 提供持續照顧的護理安老院	Nursing Home 護養院				
34	Shamshuipo 深水埗區	Hong Kong Sheng Kung Hui Welfare Council Limited 香港聖公會福利協會有限公司	Hong Kong Sheng Kung Hui Li Ka Shing Care & Attention Home for the Elderly 香港聖公會李嘉誠護理安老院@	338 Nam Cheong Street, Sham Shui Po, Kowloon 九龍深水埗南昌街338號	27761123	27765978	C&A Home Providing COC	0	0	0	257	0	**257**	M/F 男/女	Christian 基督教	Non-vegetarian 非素食
35	Shamshuipo 深水埗區	Salvation Army (The) 救世軍	Salvation Army Nam Shan Residence for Senior Citizens (The) 救世軍南山長者之家†	Units 201-232 & Units 301-332, Nam Ming House, Nam Shan Estate, Sham Shui Po, Kowloon 九龍深水埗南山邨南明樓201-232及301-332室	27775102	27779847	Conversion Home Providing COC	0	0	0	115	0	**115**	M/F 男/女	Christian 基督教	Non-vegetarian 非素食
36	Shamshuipo 深水埗區	Salvation Army (The) 救世軍	Salvation Army Nam Ming Haven for Women (The) 救世軍南明婦女之家	Units 3-16, G/F, Nam Ming House, Nam Shan Estate, Sham Shui Po, Kowloon 九龍深水埗南山邨南明樓地下3至16室	27775484	27883784	C&A Home Providing COC	0	0	0	35	0	**35**	F 女	Christian 基督教	Non-vegetarian 非素食
37	Shamshuipo 深水埗區	The Methodist Church, Hong Kong 香港基督教循道衛理聯合教會	The Methodist Church, Hong Kong Yang Memorial Methodist Social Service Sham Shui Po Nursing Home cum Day Care Service 香港基督教循道衛理聯合教會循道衛理楊震社會服務處深水埗護養院暨日間護理服務 *	2/F and 3/F, Ancillary Facilities Block, Shek Kip Mei Estate Phase 2, 100 Woh Chai Street, Sham Shui Po, Kowloon 九龍深水埗窩仔街100號石硤尾邨二期服務設施大樓二樓及三樓	23423220	23423660	Contract Home	0	0	0	6	54	**60**	M/F 男/女	Christian 基督教	Non-vegetarian 非素食
38	Shamshuipo 深水埗區	Yan Chai Hospital 仁濟醫院	Yan Chai Hospital Lee Wai Siu Kee Elderly Home 仁濟醫院李衛少琦安老院*	G/F (portion), 1/F and 2/F, Un Kin House, Un Chau Estate, 303 Un Chau Street, Sham Shui Po, Kowloon 九龍深水埗元州街303號元州邨元健樓地下（部分）、1字樓及2字樓	24811000	24817333	Contract Home	0	0	0	8	64	**72**	M/F 男/女	Nil 無	Non-vegetarian 非素食
39	Shamshuipo 深水埗區	Hong Kong Baptist Mr & Mrs Au Shue Hung Rehabilitation and Healthcare Home Limited 香港浸信會區樹洪伉儷康復護養院有限公司	Hong Kong Baptist Mr & Mrs Au Shue Hung Rehabilitation and Healthcare Home Limited 香港浸信會區樹洪伉儷康復護養院有限公司±	Parts of G/F, 1/F & 3/F and whole floors of 4/F-8/F, 55 Cornwall Street, Kowloon Tong, Kowloon 九龍九龍塘歌和老街55號地下部分、1字樓部分、3字樓部分及4至8字樓全層	27768338	23119122	NHPPS Home	0	0	0	0	112	**112**	M/F 男/女	Nil 無	Non-vegetarian 非素食
40	Shamshuipo 深水埗區	Tung Wah Group of Hospitals 東華三院	TWGHs Chu Sau Cheung Nursing Home 東華三院朱壽祥護養院*	Unit 302, 3/F and Unit 401, 4/F, Ancillary Facilities Block, Cheung Sha Wan Estate, 391 Cheung Sha Wan Road, Kowloon 九龍長沙灣道391號長沙灣邨服務設施大樓3樓302室及4樓401室	24672200	24672020	Contract Home	0	0	0	9	81	**90**	M/F 男/女	Nil 無	Non-vegetarian 非素食
41	Shamshuipo 深水埗區	Wai Ying Investment Limited 維盈投資有限公司	Evergreen (Pratas Street) Nursing Home 松悅園耆融護養院 *	Portions of G/F and M/F, 1/F and 2/F, 195 Pratas Street, Cheung Sha Wan, Kowloon 九龍長沙灣東沙島街195號地下部分、閣樓部分、1樓及2樓	26190038	26190987	Contract Home	0	0	0	8	72	**80**	M/F 男/女	Nil 無	Non-vegetarian 非素食
42	Shamshuipo 深水埗區	Ever Kind Asia Limited 永善亞洲有限公司	Alpine Nursing Home 薈耆頤養院 *	G/F (part), 1/F (part) & 2/F (part), 502 Fuk Wing Street, Sham Shui Po, Kowloon 九龍深水埗福榮街502號地下（部分）、一樓（部分）及二樓（部分）	28112602	28112502	Contract Home	0	0	0	6	54	**60**	M/F 男/女	Nil 無	Non-vegetarian 非素食
43	Shamshuipo 深水埗區	Crawfield International Limited 嘉豐國際有限公司	Ka Shui Garden Nursing Home For the Elderly 嘉瑞園護養院 *	G/F (portion), 3/F (portion) and 4/F (portion), Hoi Tat Estate Ancillary Facilities Block, 38 Sham Mong Road, Kowloon 九龍深旺道38號海達邨服務設施大樓地下（部分）、3字樓（部分）及4字樓（部分）	23939989	23939909	Contract Home	0	0	0	8	72	**80**	M/F 男/女	Nil 無	Non-vegetarian 非素食

List of Subvented, Self-financing and Contract Residential Care Homes for the Elderly Providing Subsidised Places for the Elderly (As at 30.6.2023)
提供資助安老服務宿位的津助院舍、自負盈虧院舍及合約院舍名單 (截至 30.6.2023)

S/N 編號	District 地區	Agency 機構	Name of Home 院舍名稱	Address 地址	Tel. 電話	Fax 傳真	Type of Service 服務類別	Type of Place 宿位種類					Total 總數	Sex 性別	Religion 宗教	Diet 膳食
								Hostel for the Elderly 長者宿舍	Home for the Aged 安老院	Care-and-Attention 護理安老院	Care-and-Attention Providing a Continuum-of-Care 提供持續照顧的護理安老院	Nursing Home 護養院				
44	Kowloon City 九龍城區	Asia Women's League Limited 亞洲婦女協進會有限公司	Asia Women's League Limited Chan Kwun Tung Care and Attention Home for the Elderly 亞洲婦女協進會陳昆棟頤養之家護理安老院†@	3 Hereford Road, Kowloon Tong, Kowloon 九龍九龍塘禧福道3號	23366255	23388593	Conversion Home Providing COC	0	0	0	150	0	**150**	M/F 男/女	Nil 無	Non-vegetarian 非素食
45	Kowloon City 九龍城區	Asia Women's League Limited 亞洲婦女協進會有限公司	Asia Women's League Limited Ho Leung Kit Ting Care and Attention Home for the Elderly 亞洲婦女協進會何梁潔庭頤養之家護理院@	Part of G/F, 1/F to 3/F & roof of Ho Leung Kit Ting Building, 3 Hereford Road, Kowloon Tong, Kowloon 九龍九龍塘禧福道3號何梁潔庭大樓地下部分、1字樓至3字樓及頂樓	23376603	23372204	C&A Home Providing COC	0	0	0	120	0	**120**	M/F 男/女	Nil 無	Non-vegetarian 非素食
46	Kowloon City 九龍城區	Tung Wah Group of Hospitals 東華三院	TWGHs Wong Cho Tong Care and Attention Home 東華三院黃祖棠護理安老院@	1/F-5/F, TWGHs Wong Cho Tong Social Service Building, 39 Sheung Shing Street, Ho Man Tin, Kowloon 九龍何文田常盛街39號東華三院黃祖棠社會服務大樓1字樓至5字樓	27155933	27125229	C&A Home Providing COC	0	0	0	278	0	**278**	M/F 男/女	Nil 無	Non-vegetarian 非素食
47	Kowloon City 九龍城區	Po Leung Kuk 保良局	Po Leung Kuk Merry Court for the Senior 保良局壬午年耆樂居*	G/F and 1/F, Choi Man House and Yee Man House, Ho Man Tin Estate, Kowloon 九龍何文田邨采文樓及綺文樓地下及一樓	22421713	22421579	Contract Home	0	0	0	11	99	**110**	M/F 男/女	Nil 無	Non-vegetarian 非素食
48	Kowloon City 九龍城區	Neighbourhood Advice-Action Council (The) 鄰舍輔導會	Neighbourhood Advice-Action Council Shanghai Fraternity Association Care & Attention Home for the Elderly (The) 鄰舍輔導會上海總會護理安老院	Part of Level 4 and part of Level of 5 of Yan Man House, Ho Man Tin Estate and Ko Fai House, Kwun Fai Court, Ho Man Tin, Kowloon 九龍何文田邨欣文樓四樓（部分）和五樓（部分）及冠暉苑高暉閣四樓（部分）和五樓（部分）	22420311	22420211	C&A Home Providing COC	0	0	0	100	0	**100**	M/F 男/女	Nil 無	Non-vegetarian 非素食
49	Yau Tsim Mong 油尖旺區	Salvation Army (The) 救世軍	Salvation Army Hoi Tai Residence for Senior Citizens (The) 救世軍海泰長者之家	2/F, Hoi Tai House, Hoi Fu Court, Hoi Ting Road, Mong Kok West, Kowloon 九龍旺角西海庭道海富苑海泰閣2字樓	21482000	26269992	C&A Home Providing COC	0	0	0	98	0	**98**	M/F 男/女	Christian 基督教	Non-vegetarian 非素食
50	Yau Tsim Mong 油尖旺區	Po Leung Kuk 保良局	Po Leung Kuk Tai Kok Tsui Home for the Elderly cum Cherish Day Care Centre for the Elderly 保良局大角咀護老院暨耆順長者日間護理中心 *	3/F and 5/F, 9 Tai Kok Tsui Road, Kowloon 九龍大角咀道9號三樓及五樓	27963766	27963722	Contract Home	0	0	0	5	51	**56**	M/F 男/女	Nil 無	Non-vegetarian 非素食
51	Yau Tsim Mong 油尖旺區	Tung Wah Group of Hospitals 東華三院	TWGHs Willow Lodge 東華三院翠柳頤庭*	Parts of G/F, 1/F, 2/F and 3/F, 18 Willow Street, Tai Kok Tsui, Kowloon 九龍大角咀柳樹街18號~~2樓及~~地下（部分）、1樓（部分）、2樓（部分）及3樓（部分）	28056673	28056556	Contract Home	0	0	0	6	55	**61**	M/F 男/女	Nil 無	Non-vegetarian 非素食
52	Yau Tsim Mong 油尖旺區	Lok Sin Tong Benevolent Society, Kowloon (The) 九龍樂善堂	Lok Sin Tong Hoi Wang Road Nursing Home 樂善堂海泓道護養院*	2/F and 3/F, 1 Hoi Wang Road, South West Kowloon, Kowloon 九龍西南九龍海泓道1號2及3樓	23076422	23076433	Contract Home	0	0	0	6	55	**61**	M/F 男/女	Nil 無	Non-vegetarian 非素食
53	Yau Tsim Mong 油尖旺區	Evangelical Lutheran Church of Hong Kong 基督教香港信義會社會服務部	ELCHK, Serene Court 基督教香港信義會恩海居*	G/F, UG/F and 1/F, 12 Hoi Fai Road, Kowloon 九龍海輝道12號地下、高層地下及1字樓	24898000	24817222	Contract Home	0	0	0	9	85	**94**	M/F 男/女	Nil 無	Non-vegetarian 非素食
54	Wong Tai Sin 黃大仙區	Wai Ying Investment Limited 維盈投資有限公司	Evergreen (Tsz Ching) Nursing Home cum Day Care Centre 松悅園耆逸護養院暨日間護理中心*	3/F and 4/F, Ancillary Facilities Block, Tsz Ching Estate, Wong Tai Sin, Kowloon 九龍黃大仙慈正邨服務設施大樓3字樓及4字樓	26101775	26101738	Contract Home	0	0	0	10	92	**102**	M/F 男/女	Nil 無	Non-vegetarian 非素食
55	Wong Tai Sin 黃大仙區	Helping Hand 伸手助人協會	Helping Hand Hong Kong Bank Foundation Lok Fu Care Home 伸手助人協會匯豐銀行基金樂富護老院	G/F, Lok Man House, Lok Fu Estate, Wong Tai Sin, Kowloon 九龍黃大仙樂富邨樂民樓地下	23360716	23046472	C&A Home Providing COC	0	0	0	75	0	**75**	M/F 男/女	Nil 無	Non-vegetarian 非素食

List of Subvented, Self-financing and Contract Residential Care Homes for the Elderly Providing Subsidised Places for the Elderly (As at 30.6.2023)
提供資助安老服務宿位的津助院舍、自負盈虧院舍及合約院舍名單 (截至 30.6.2023)

S/N<br>編號	District<br>地區	Agency<br>機構	Name of Home<br>院舍名稱	Address<br>地址	Tel.<br>電話	Fax<br>傳真	Type of Service<br>服務類別	Type of Place<br>宿位種類					Total<br>總數	Sex<br>性別	Religion<br>宗教	Diet<br>膳食
								Hostel for the Elderly<br>長者宿舍	Home for the Aged<br>安老院	Care-and-Attention<br>護理安老院	Care-and-Attention Providing a Continuum-of-Care<br>提供持續照顧的護理安老院	Nursing Home<br>護養院				
56	Wong Tai Sin<br>黃大仙區	Chi Lin Nunnery<br>志蓮淨苑	Chi Lin Nunnery Chi Lin Care and Attention Home<br>志蓮淨苑志蓮護理安老院@	1/F to 4/F, 5 Chi Lin Drive, Diamond Hill, Kowloon<br>九龍鑽石山志蓮道5號1字樓至4字樓	23541853	23231939	Conversion Home Providing COC	0	0	0	306	0	**306**	M/F<br>男/女	Buddhist<br>佛教	Vegetarian<br>素食
57	Wong Tai Sin<br>黃大仙區	Tung Wah Group of Hospitals<br>東華三院	TWGHs Ho Tung Home for the Elderly<br>東華三院何東安老院†	Rooms 201-233 and 235, Fu Yan House, Fu Shan Estate, Diamond Hill, Kowloon<br>九龍鑽石山富山邨富仁樓201至233及235室	23512953	23229799	Conversion Home Providing COC	0	0	0	60	0	**60**	M/F<br>男/女	Nil<br>無	Non-vegetarian<br>非素食
58	Wong Tai Sin<br>黃大仙區	Hong Kong Sheng Kung Hui Welfare Council Limited<br>香港聖公會福利協會有限公司	Hong Kong Sheng Kung Hui Nursing Home<br>香港聖公會護養院	6 Chun Yan Street, Wong Tai Sin, Kowloon<br>九龍黃大仙親仁街6號	23255330	23255377	Nursing Home	0	0	0	0	280	**280**	M/F<br>男/女	Christian<br>基督教	Vegetarian/ Non-vegetarian<br>素食/ 非素食
59	Wong Tai Sin<br>黃大仙區	The Lutheran Church - Hong Kong Synod Limited<br>香港路德會有限公司	Hong Kong Lutheran Social Service Fung Tak Lutheran Home for the Elderly<br>香港路德會社會服務處路德會鳳德安老院†@	G/F & NDF2 of Ban Fung House & Ngan Fung House and Flat 133 &134, Ngan Fung House, Fung Tak Estate, Diamond Hill, Kowloon<br>九龍鑽石山鳳德邨斑鳳樓、銀鳳樓地下及二樓及銀鳳樓133及134室	23260206	23206160	Conversion Home Providing COC	0	0	0	106	0	**106**	M/F<br>男/女	Christian<br>基督教	Non-vegetarian<br>非素食
60	Wong Tai Sin<br>黃大仙區	Pok Oi Hospital<br>博愛醫院	Pok Oi Hospital Chan Feng Men Ling Care and Attention Home<br>博愛醫院陳馮曼玲護理安老院@	G/F & 1/F, Lok Shing House & Lok Wong House, Tsz Lok Estate, Tsz Wan Shan, Kowloon<br>九龍慈雲山慈樂邨樂誠樓及樂旺樓地下及一樓	23263319	23298870	C&A Home Providing COC	0	0	0	156	0	**156**	M/F<br>男/女	Nil<br>無	Non-vegetarian<br>非素食
61	Wong Tai Sin<br>黃大仙區	Sik Sik Yuen<br>嗇色園	Ho Yam Care and Attention Home for the Elderly (Sponsored by Sik Sik Yuen)<br>嗇色園主辦可蔭護理安老院@	G/F (portion) and 2/F to 6/F, 38 Fung Tak Road, Tsz Wan Shan, Kowloon<br>九龍慈雲山鳳德道38號地下（部分）及2字樓至6字樓	23215580	21485621	C&A Home Providing COC	0	0	0	272	0	**272**	M/F<br>男/女	Buddhist/ Taoist/ Confucian<br>佛/道/孔教	Non-vegetarian<br>非素食
62	Wong Tai Sin<br>黃大仙區	Lok Sin Tong Benevolent Society, Kowloon (The)<br>九龍樂善堂	Lok Sin Tong Leung Kau Kui Home for the Elderly<br>樂善堂梁銶琚敬老之家†	2/F, Wah Yuen House, Chuk Yuen South Estate, Wong Tai Sin, Kowloon<br>九龍黃大仙竹園南邨華園樓2字樓	27260405	23514921	Conversion Home Providing COC	0	0	0	87	0	**87**	M/F<br>男/女	Nil<br>無	Non-vegetarian<br>非素食
63	Wong Tai Sin<br>黃大仙區	E.T. Investment Limited<br>頤盈投資有限公司	Oasis Nursing Home<br>紫雲間沁怡護養院 *	LG/2 (part), G/F (part), 1/F to 6/F and 7/F (part), Lok Foon House, Tsz Lok Estate, Tsz Wan Shan, Kowloon<br>九龍慈雲山慈樂邨樂歡樓低層2字樓（部分）、地下（部分）、1字樓至6字樓及7字樓（部分）	21550303	21552911	Contract Home	0	0	0	10	96	**106**	M/F<br>男/女	Nil<br>無	Non-vegetarian<br>非素食
64	Sai Kung<br>西貢區	Tung Sin Tan<br>通善壇	Tung Sin Tan Home for the Aged<br>通善壇安老院†	D.D. 217 Lot 1119, Off Hiram's Highway, Hebe Haven, Sai Kung, New Territories (same as Man Kung Wo Road, Habitat, Pak Sha Wan, Sai Kung, New Territories)<br>新界西貢白沙灣近西貢公路丈量約份第217約地段第1119號（即西貢白沙灣白沙台孟公窩路）	27198675	27198844	Conversion Home Providing COC	0	0	0	50	0	**50**	F<br>女	Taoist<br>道教	Non-vegetarian<br>非素食
65	Sai Kung<br>西貢區	Helping Hand<br>伸手助人協會	Helping Hand Father Sean Burke Care Home for the Elderly<br>伸手助人協會畢尚華神父護老頤養院	Nin Wah Road, Cheung Muk Tau North, Sai Kung, New Territiries<br>新界西貢樟木頭北年華路	21449969	21449906	C&A Home Providing COC	0	0	0	211	0	**211**	M/F<br>男/女	Nil<br>無	Non-vegetarian<br>非素食
66	Sai Kung<br>西貢區	Hong Kong Society for the Aged (The)<br>香港耆康老人福利會	SAGE Tung Lin Kok Yuen Home for the Elderly<br>耆康會東蓮覺苑護理安老院†	4/F, Hong Lam House and On Lam House, Tsui Lam Estate, Tseung Kwan O, Kowloon<br>九龍將軍澳翠林邨康林樓及安林樓4字樓	27020066	21748043	Conversion Home Providing COC	0	0	0	112	0	**112**	M/F<br>男/女	Nil<br>無	Non-vegetarian<br>非素食

List of Subvented, Self-financing and Contract Residential Care Homes for the Elderly Providing Subsidised Places for the Elderly (As at 30.6.2023)
提供資助安老服務宿位的津助院舍、自負盈虧院舍及合約院舍名單 (截至 30.6.2023)

S/N<br>編號	District<br>地區	Agency<br>機構	Name of Home<br>院舍名稱	Address<br>地址	Tel.<br>電話	Fax<br>傳真	Type of Service<br>服務類別	Type of Place<br>宿位種類					Total<br>總數	Sex<br>性別	Religion<br>宗教	Diet<br>膳食
								Hostel for the Elderly<br>長者宿舍	Home for the Aged<br>安老院	Care-and-Attention<br>護理安老院	Care-and-Attention Providing a Continuum-of-Care<br>提供持續照顧的護理安老院	Nursing Home<br>護養院				
67	Sai Kung<br>西貢區	Hong Kong Sheng Kung Hui Welfare Council Limited<br>香港聖公會福利協會有限公司	Hong Kong Sheng Kung Hui<br>John Yuen Home for the Elderly<br>香港聖公會阮維揚長者之家@	Portion of G/F, and 1/F, 2/F and 3/F, Hong Kong Sheng Kung Hui Tseung Kwan O Aged Care Complex, 101 Po Lam Road North, Tseung Kwan O, Kowloon<br>九龍將軍澳寶琳北路101號香港聖公會將軍澳安老服務大樓地下（部分）、1樓、2樓及3樓	27029897	27021622	C&A Home Providing COC	0	0	0	186	0	**186**	M/F<br>男/女	Christian<br>基督教	Non-vegetarian<br>非素食
68	Sai Kung<br>西貢區	The Lutheran Church-Hong Kong Synod Limited<br>香港路德會有限公司	Hong Kong Lutheran Social Service, LC-HKS<br>Mr. & Mrs. Lawrence Wong Second Lutheran Home for the Elderly<br>香港路德會社會服務處路德會黃鎮林伉儷第二安老院†	G/F & 1/F, Po Chung House & Po Pak House, Po Ming Court, Tseung Kwan O, Kowloon<br>九龍將軍澳寶明苑寶松閣、寶柏閣地下及一樓	21782992	21782994	Conversion Home Providing COC	0	0	0	114	0	**114**	M/F<br>男/女	Christian<br>基督教	Non-vegetarian<br>非素食
69	Sai Kung<br>西貢區	Christian Family Service Centre<br>基督教家庭服務中心	Christian Family Service Centre Yang Chen House<br>基督教家庭服務中心養真苑†	G/F and 1/F, Tak Hong House and Tak Chi House, Hau Tak Estate, Tseung Kwan O, Kowloon<br>九龍將軍澳厚德邨德康樓及德志樓地下及一樓	27063018	27063161	Conversion Home Providing COC	0	0	0	122	0	**122**	M/F<br>男/女	Christian<br>基督教	Non-vegetarian<br>非素食
70	Sai Kung<br>西貢區	Christian Family Service Centre<br>基督教家庭服務中心	Christian Family Service Centre Yam Pak Charitable Foundation King Lam Home for the Elderly<br>基督教家庭服務中心任白慈善基金景林安老院†	G/F & 1/F, King Min House, King Lam Estate, Tseung Kwan O, Kowloon<br>九龍將軍澳景林邨景棉樓地下及1字樓	27018655	27061922	Conversion Home Providing COC	0	0	0	103	0	**103**	M/F<br>男/女	Christian<br>基督教	Non-vegetarian<br>非素食
71	Sai Kung<br>西貢區	Haven of Hope Christian Service<br>基督教靈實協會	Haven of Hope Christian Service<br>Haven of Hope Nursing Home<br>基督教靈實協會靈實護養院	23 Haven of Hope Road, Tseung Kwan O, Kowloon<br>九龍將軍澳靈實路23號	27032100	27032111	Nursing Home	0	0	0	0	270	**270**	M/F<br>男/女	Christian<br>基督教	Non-vegetarian<br>非素食
72	Sai Kung<br>西貢區	Salvation Army (The)<br>救世軍	Salvation Army Po Lam Residence for Senior Citizens (The)<br>救世軍寶林長者之家†@	4/F, Po Kan House, Po Lam Estate, Tseung Kwan O, Kowloon<br>九龍將軍澳寶林邨寶勤樓4字樓	27015828	26232500	Conversion Home Providing COC	0	0	0	105	0	**105**	M/F<br>男/女	Christian<br>基督教	Non-vegetarian<br>非素食
73	Sai Kung<br>西貢區	Haven of Hope Christian Service<br>基督教靈實協會	Haven of Hope Sister Annie Skau Holistic Care Centre<br>靈實司務道寧養院± #	19-21 Haven of Hope Road, Tseung Kwan O, Kowloon<br>九龍將軍澳靈實路19-21號	27033000	27035575	NHPPS Home	0	0	0	0	3	**3**	M/F<br>男/女	Christian<br>基督教	Non-vegetarian<br>非素食
74	Kwun Tong<br>觀塘區	Kowloon Women's Welfare Club (The)<br>九龍婦女福利會	Kowloon Women's Welfare Club Wong Cheung Kin Memorial Hostel for the Elderly (The)<br>九龍婦女福利會黃張見紀念老人之家†@	Rooms 201-260, Floor 2, Tin Hang House, Shun Tin Estate, Kwun Tong, Kowloon<br>九龍觀塘順天邨天衡樓2字樓201-260室	27972556	23429217	Conversion Home Providing COC	0	0	0	93	0	**93**	M/F<br>男/女	Nil<br>無	Non-vegetarian<br>非素食
75	Kwun Tong<br>觀塘區	Po Leung Kuk<br>保良局	Po Leung Kuk Siu Ming Memorial Home cum Care & Attention Unit<br>保良局蕭明紀念護老院†@	G/F and 1/F, Kwong Hin House and Kwong Ngar House, Kwong Tin Estate, Lam Tin, Kowloon<br>九龍藍田廣田邨廣軒樓及廣雅樓地下及1字樓	23494800	29529867	Conversion Home Providing COC	0	0	0	137	0	**137**	M/F<br>男/女	Nil<br>無	Non-vegetarian<br>非素食
76	Kwun Tong<br>觀塘區	Hong Kong Chinese Women's Club (The)<br>香港中國婦女會	Hong Kong Chinese Women's Club Madam Wong Chan Sook Ying Memorial Care and Attention Home for the Aged (The)<br>香港中國婦女會黃陳淑英紀念護理安老院†@	6 Pik Wan Road, Yau Tong, Kowloon<br>九龍油塘碧雲道6號	27171351	23468591	Conversion Home Providing COC	0	0	0	214	0	**214**	M/F<br>男/女	Nil<br>無	Non-vegetarian<br>非素食
77	Kwun Tong<br>觀塘區	Hong Kong Buddhist Association (The)<br>香港佛教聯合會	Buddhist Sum Ma Shui Ying Care & Attention Home for the Elderly<br>佛教沈馬瑞英護理安老院†@	8 Kung Lok Road, Kwun Tong, Kowloon<br>九龍觀塘功樂道8號	27637838	27637264	Conversion Home Providing COC	0	0	0	217	0	**217**	M/F<br>男/女	Buddhist<br>佛教	Vegetarian/<br>Non-vegetarian<br>素食/<br>非素食

List of Subvented, Self-financing and Contract Residential Care Homes for the Elderly Providing Subsidised Places for the Elderly (As at 30.6.2023)
提供資助安老服務宿位的津助院舍、自負盈虧院舍及合約院舍名單 (截至 30.6.2023)

S/N<br>編號	District<br>地區	Agency<br>機構	Name of Home<br>院舍名稱	Address<br>地址	Tel.<br>電話	Fax<br>傳真	Type of Service<br>服務類別	Type of Place 宿位種類					Total<br>總數	Sex<br>性別	Religion<br>宗教	Diet<br>膳食
								Hostel for the Elderly<br>長者宿舍	Home for the Aged<br>安老院	Care-and-Attention<br>護理安老院	Care-and-Attention Providing a Continuum-of-Care<br>提供持續照顧的護理安老院	Nursing Home<br>護養院				
78	Kwun Tong<br>觀塘區	Hong Kong Society for the Aged (The)<br>香港耆康老人福利會	SAGE Kai Yip Home for the Elderly<br>耆康會啟業護理安老院†	Unit 201 to 227, 2/F, Kai Shing House, Kai Yip Estate, Kowloon Bay, Kowloon<br>九龍九龍灣啟業邨啟盛樓2字樓201至227室	27552392	27507179	Conversion Home Providing COC	0	0	0	53	0	**53**	M/F<br>男/女	Nil<br>無	Non-vegetarian<br>非素食
79	Kwun Tong<br>觀塘區	Hong Kong Society for the Aged (The)<br>香港耆康老人福利會	SAGE Mrs Y. K. Fung Home for the Elderly<br>耆康會馮堯敬夫人護理安老院†	2/F, Ancillary Facilities Block, Ping Tin Estate, Lam Tin, Kowloon<br>九龍藍田平田邨服務設施大樓2字樓	27099917	27099962	Conversion Home Providing COC	0	0	0	87	0	**87**	M/F<br>男/女	Nil<br>無	Non-vegetarian<br>非素食
80	Kwun Tong<br>觀塘區	Hong Kong Christian Service<br>香港基督教服務處	Hong Kong Christian Service Shun Lee Home for the Elderly<br>香港基督教服務處順利安老院†	4/F, Lee Foo House, Shun Lee Estate, Kwun Tong, Kowloon<br>九龍觀塘順利邨利富樓4字樓	23420346	27934238	Conversion Home Providing COC	0	0	0	58	0	**58**	M/F<br>男/女	Christian<br>基督教	Non-vegetarian<br>非素食
81	Kwun Tong<br>觀塘區	Hong Kong Sheng Kung Hui Welfare Council Limited<br>香港聖公會福利協會有限公司	Hong Kong Sheng Kung Hui<br>Good Shepherd Home for the Elderly<br>香港聖公會牧愛長者之家†@	Unit 141 to 156 and 201 to 260, Fai Wah House, Lok Wah South Estate, Ngau Tau Kok, Kowloon<br>九龍牛頭角樂華南邨輝華樓141至156室及201至260室	27593378	27577885	Conversion Home Providing COC	0	0	0	115	0	**115**	M/F<br>男/女	Christian<br>基督教	Non-vegetarian<br>非素食
82	Kwun Tong<br>觀塘區	Salvation Army (The)<br>救世軍	Salvation Army Tak Tin Residence for Senior Citizens (The)<br>救世軍德田長者之家†@	2/F, Tak King House, Tak Tin Estate, Lam Tin, Kowloon<br>九龍藍田德田邨德敬樓2字樓	23478183	23799539	Conversion Home Providing COC	0	0	0	67	0	**67**	M/F<br>男/女	Christian<br>基督教	Non-vegetarian<br>非素食
83	Kwun Tong<br>觀塘區	Alice Ho Miu Ling Nethersole Charity Foundation<br>雅麗氏何妙齡那打素慈善基金會	Alice Ho Miu Ling Nethersole Nursing Home<br>雅麗氏何妙齡那打素護養院	2-6/F & 7/F(Part), Kowloon Bay Health Centre, 9 Kai Yan Street, Kowloon Bay, Kowloon<br>九龍九龍灣啟仁街9號九龍灣健康中心二至七樓部分	21167300	21160070	Nursing Home	0	0	0	0	238	**238**	M/F<br>男/女	Christian<br>基督教	Non-vegetarian<br>非素食
84	Kwun Tong<br>觀塘區	Ever Kind Asia Limited<br>永善亞洲有限公司	Altru Nursing Home<br>恩耆頤養院*	4/F, Ancillary Facilities Block, Sau Mau Ping Estate, Kwun Tong, Kowloon<br>九龍觀塘秀茂坪邨服務設施大樓四樓	2650 3000	2650 3080	Contract Home	0	0	0	10	86	**96**	M/F<br>男/女	Nil<br>無	Non-vegetarian<br>非素食
85	Kwun Tong<br>觀塘區	Grace Healthcare Limited<br>頌恩醫療護理有限公司	Grace Nursing Home (Tak Tin)<br>頌恩護理院（德田）*	4/F to 6/F, Tak Yan House, Tak Tin Estate, Lam Tin, Kowloon<br>九龍藍田德田邨德欣樓四樓至六樓	31571368	31570350	Contract Home	0	0	0	4	38	**42**	M/F<br>男/女	Nil<br>無	Non-vegetarian<br>非素食
86	Kwun Tong<br>觀塘區	Hong Kong Sheng Kung Hui Welfare Council Limited<br>香港聖公會福利協會有限公司	Hong Kong Sheng Kung Hui Home of Loving Care for the Elderly<br>香港聖公會恩慈長者之家†@	1/F to 3/F, Tak Yan House, Tak Tin Estate, Lam Tin, Kowloon<br>九龍藍田德田邨德欣樓1字樓至3字樓	26547744	26572683	Conversion Home Providing COC	0	0	0	102	0	**102**	M/F<br>男/女	Christian<br>基督教	Non-vegetarian<br>非素食
87	Kwun Tong<br>觀塘區	Yuen Yuen Institute (The)<br>圓玄學院	Yuen Yuen Nursing Home cum Day Care Centre for the Elderly (Shun Lee Estate)<br>圓玄護養院暨長者日間護理中心（順利邨）*	Unit A on 2/F, 3/F and 4/F of Shun Lee Shopping Centre Phase II, Shun Lee Estate, Kowloon<br>九龍順利邨順利商場二期二樓A室及三至四樓	23411061	23411091	Contract Home	0	0	0	6	60	**66**	M/F<br>男/女	Buddhist/ Taoist/ Confucian<br>佛/道/孔教	Non-vegetarian<br>非素食
88	Kwun Tong<br>觀塘區	Wai Ying Investment Limited<br>維盈投資有限公司	Evergreen (On Tai) Nursing Home Cum Day Care Centre<br>松悅園耆泰護養院暨日間護理中心*	1/F (portion) and 2/F (portion), On Tai Estate Ancillary Facilities Block, 23 On Sau Road, Kwun Tong, Kowloon<br>九龍觀塘安秀道23號安泰邨服務設施大樓一樓（部分）及二樓（部分）	26190039	26190313	Contract Home	0	0	0	8	72	**80**	M/F<br>男/女	Nil<br>無	Non-vegetarian<br>非素食
89	Shatin<br>沙田區	The Chinese Rhenish Church Hong Kong Synod<br>中華基督教禮賢會香港區會	The Chinese Rhenish Church Hong Kong Synod Wong Siu Ching Rhenish Home For The Elderly<br>中華基督教禮賢會香港區會禮賢會王少清頤養院†	Portion of G/F, and 1/F, Green Heron House, Sha Kok Estate, Sha Tin, New Territories<br>新界沙田沙角邨綠鷺樓地下（部分）及二樓	26479291	26450547	Conversion Home Providing COC	0	0	0	79	0	**79**	M/F<br>男/女	Christian<br>基督教	Non-vegetarian<br>非素食

List of Subvented, Self-financing and Contract Residential Care Homes for the Elderly Providing Subsidised Places for the Elderly (As at 30.6.2023)
提供資助安老服務宿位的津助院舍、自負盈虧院舍及合約院舍名單 (截至 30.6.2023)

S/N 編號	District 地區	Agency 機構	Name of Home 院舍名稱	Address 地址	Tel. 電話	Fax 傳真	Type of Service 服務類別	Type of Place 宿位種類: Hostel for the Elderly 長者宿舍	Type of Place 宿位種類: Home for the Aged 安老院	Type of Place 宿位種類: Care-and-Attention 護理安老院	Type of Place 宿位種類: Care-and-Attention Providing a Continuum-of-Care 提供持續照顧的護理安老院	Type of Place 宿位種類: Nursing Home 護養院	Total 總數	Sex 性別	Religion 宗教	Diet 膳食
90	Shatin 沙田區	Yan Chai Hospital 仁濟醫院	Yan Chai Hospital Li Chan Yuk Sim Elderly Home 仁濟醫院李陳玉嬋安老院†	Units 1 to 10 of G/F and Units 101 to 124 of 1/F, Pok Chi House, Pok Hong Estate, Sha Tin, New Territories 新界沙田博康邨博智樓地下1至10室及1字樓101至124室	26463022	26379935	Conversion Home Providing COC	0	0	0	90	0	**90**	M/F 男/女	Nil 無	Non-vegetarian 非素食
91	Shatin 沙田區	Tung Wah Group of Hospitals 東華三院	TWGHs Ma Hing Chou Home for the Elderly 東華三院馬興秋安老院†	Units 601-640 and 620A, Fook Hoi House, Lek Yuen Estate, Sha Tin, New Territories 新界沙田瀝源邨福海樓601-640室及620A室	26919363	26081412	Conversion Home Providing COC	0	0	0	75	0	**75**	M/F 男/女	Nil 無	Non-vegetarian 非素食
92	Shatin 沙田區	Tung Wah Group of Hospitals 東華三院	TWGHs Mok Wong Fung Yee Home for the Elderly 東華三院莫黃鳳儀安老院†	Units 1 to 60, 3/F, Sun Yee House, Sun Chui Estate, Sha Tin, New Territories 新界沙田新翠邨新儀樓3字樓1至60室	26042293	26080715	Conversion Home Providing COC	0	0	0	75	0	**75**	M/F 男/女	Nil 無	Non-vegetarian 非素食
93	Shatin 沙田區	Tung Wah Group of Hospitals 東華三院	TWGHs Lo Man Huen Home for the Elderly 東華三院羅文壎安老院†	Units 201-240, Mei Yeung House, Mei Lam Estate, Sha Tin, New Territories 新界沙田美林邨美楊樓201至240室	26057172	26057169	Conversion Home Providing COC	0	0	0	60	0	**60**	M/F 男/女	Nil 無	Non-vegetarian 非素食
94	Shatin 沙田區	Tung Wah Group of Hospitals 東華三院	TWGHs Chan Han Home for the Elderly 東華三院陳嫺安老院†@	Units 101 to 108, 117 to 124 & 201 to 224, Yiu Him House, Yiu On Estate, Ma On Shan, New Territories 新界馬鞍山耀安邨耀謙樓101至108室、117至124室及201至224室	26415582	26434937	Conversion Home Providing COC	0	0	0	88	0	**88**	M/F 男/女	Nil 無	Non-vegetarian 非素食
95	Shatin 沙田區	Tung Wah Group of Hospitals 東華三院	TWGHs Lo Wong Yuk Man Nursing Home cum Day Care Centre 東華三院羅王玉文護養院暨日間中心*	Carpark Floor (part) and Carpark Floor High Level, Tai Wai Social Service Building, 1 Mei Tin Road, Tai Wai, Sha Tin, New Territories 新界沙田大圍美田路１號大圍社會服務大樓停車場層（部分）及停車場高層	23505200	23505618	Contract Home	0	0	0	6	55	**61**	M/F 男/女	Nil 無	Non-vegetarian 非素食
96	Shatin 沙田區	Caritas - Hong Kong 香港明愛	Caritas Harold H.W. Lee Care and Attention Home 明愛利孝和護理安老院@	17 Kong Pui Street, Sha Tin, New Territories 新界沙田崗背街17號	21642400	21642426	C&A Home Providing COC	0	0	0	228	0	**228**	M/F 男/女	Catholic 天主教	Non-vegetarian 非素食
97	Shatin 沙田區	Hong Kong Society for the Aged (The) 香港耆康老人福利會	SAGE Kwan Fong Nim Chee Home for the Elderly 耆康會群芳念慈護理安老院@	27 Chap Wai Kon Street, Sha Tin, New Territories 新界沙田插桅杆街27號	26370488	26361091	C&A Home Providing COC	0	0	0	204	0	**204**	M/F 男/女	Nil 無	Non-vegetarian 非素食
98	Shatin 沙田區	Hong Kong Society for the Aged (The) 香港耆康老人福利會	SAGE Mrs. Wong Yee Jar Jat Memorial Home for the Elderly 耆康會王余家潔紀念護理安老院†	1/F and 2 Wings on G/F, Hin Yeung House, Hin Keng Estate, Sha Tin, New Territories 新界沙田顯徑邨顯揚樓地下兩翼及二樓	26010332	26071606	Conversion Home Providing COC	0	0	0	89	0	**89**	M/F 男/女	Nil 無	Non-vegetarian 非素食
99	Shatin 沙田區	Hong Kong Sheng Kung Hui Welfare Council Limited 香港聖公會福利協會有限公司	Hong Kong Sheng Kung Hui St Paul's Home for the Elderly 香港聖公會保羅長者之家†	Units 106-113 and 201-227, Fung Wai House, Sun Tin Wai Estate, Sha Tin, New Territories 新界沙田新田圍邨豐圍樓106-113及201-227室	26973363	26932561	Conversion Home Providing COC	0	0	0	80	0	**80**	M/F 男/女	Christian 基督教	Non-vegetarian 非素食
100	Shatin 沙田區	Salvation Army (The) 救世軍	Salvation Army Lung Hang Residence for Senior Citizens (The) 救世軍隆亨長者之家†	Units 341 to 360 and 421 to 460, Wing Sam House, Lung Hang Estate, Sha Tin, New Territories 新界沙田隆亨邨榮心樓341至360及421至460室	26023696	26935630	Conversion Home Providing COC	0	0	0	60	0	**60**	M/F 男/女	Christian 基督教	Non-vegetarian 非素食
101	Shatin 沙田區	Salvation Army (The) 救世軍	Salvation Army Bradbury Home of Loving Kindness (The) 救世軍白普理慈愛長者之家@	16 Tung Lo Wan Hill Road, Tai Wai, Sha Tin, New Territories 新界沙田大圍銅鑼灣山路16號	26015000	26040601	C&A Home Providing COC	0	0	0	136	0	**136**	M/F 男/女	Christian 基督教	Non-vegetarian 非素食

List of Subvented, Self-financing and Contract Residential Care Homes for the Elderly Providing Subsidised Places for the Elderly (As at 30.6.2023)
提供資助安老服務宿位的津助院舍、自負盈虧院舍及合約院舍名單 (截至 30.6.2023)

S/N 編號	District 地區	Agency 機構	Name of Home 院舍名稱	Address 地址	Tel. 電話	Fax 傳真	Type of Service 服務類別	Type of Place 宿位種類<br>Hostel for the Elderly 長者宿舍	Home for the Aged 安老院	Care-and-Attention 護理安老院	Care-and-Attention Providing a Continuum-of-Care 提供持續照顧的護理安老院	Nursing Home 護養院	Total 總數	Sex 性別	Religion 宗教	Diet 膳食
102	Shatin 沙田區	Sik Sik Yuen 嗇色園	Ho Shing Home for the Elderly (Sponsored by Sik Sik Yuen) 嗇色園主辦可誠護理安老院†@	G/F & 1/F, Alder House, Kwong Yuen Estate, Sha Tin, New Territories 新界沙田廣源邨廣楊樓地下及一樓	26354262	26492359	Conversion Home Providing COC	0	0	0	90	0	**90**	M/F 男/女	Buddhist/ Taoist/ Confucian 佛/道/孔教	Non-vegetarian 非素食
103	Shatin 沙田區	Care & Services Company Limited 嘉頤護理有限公司	Grand Residence 嘉頤薈*	LG/F (Portion) and Unit 1 on L1/F, Ming Chuen House, Shui Chuen O Estate, Sha Tin, New Territories 新界沙田水泉澳邨明泉樓L1層1室及地下低層（部分）	26064600	26064328	Contract Home	0	0	0	6	54	**60**	M/F 男/女	Nil 無	Non-vegetarian 非素食
104	Shatin 沙田區	On Fuk Nursing Home Limited 安福護老院有限公司	Sino Chun Yeung Nursing Home 華創駿洋居*	101, 1/F, Chun Wu House, Chun Yeung Estate, Fotan, New Territories, Hong Kong 香港新界火炭駿洋邨駿湖樓一樓101室	27890899	27890029	Contract Home	0	0	0	7	63	**70**	M/F 男/女	Nil 無	Non-vegetarian 非素食
105	Shatin 沙田區	Po Leung Kuk 保良局	Po Leung Kuk Shek Mun Home cum Day Care Centre for the Elderly 保良局碩門護老院暨長者日間護理中心*	G/F (portion), and 1/F to 4/F, Shek Mun Estate Social Service Building, 18 On Muk Street, Shek Mun, Sha Tin, New Territories 新界沙田石門安睦街18號碩門邨社會服務大樓地下（部分）及一至四樓	25485800	28027866	Contract Home	0	0	0	9	81	**90**	M/F 男/女	Nil 無	Non-vegetarian 非素食
106	Tai Po 大埔區	Lok Sin Tong Benevolent Society, Kowloon (The) 九龍樂善堂	Lok Sin Tong Chu Ting Cheong Home for the Aged 樂善堂朱定昌頤養院†@	8 Wan Tau Street, Tai Po Market, Tai Po, New Territories 新界大埔大埔墟運頭街8號	26586160	26503060	Conversion Home Providing COC	0	0	0	222	0	**222**	M/F 男/女	Nil 無	Non-vegetarian 非素食
107	Tai Po 大埔區	Tung Wah Group of Hospitals 東華三院	TWGHs Hui Lai Kuen Home for the Elderly 東華三院許麗娟安老院†@	G/F and 1/F, Tai Ling House, Tai Yuen Estate, Tai Po, New Territories 新界大埔大元邨泰寧樓地下及1字樓	26671803	26607202	Conversion Home Providing COC	0	0	0	75	0	**75**	M/F 男/女	Nil 無	Non-vegetarian 非素食
108	Tai Po 大埔區	Tung Wah Group of Hospitals 東華三院	TWGHs Wu York Yu Care and Attention Home 東華三院伍若瑜護理安老院@	Lot 152 in DD 27 (also known as 93 Sam Mun Tsai Road), Tai Po, New Territories 新界大埔丈量第27約地段第152號 (亦稱三門仔路93號)	26605388	26623780	C&A Home Providing COC	0	0	0	255	0	**255**	M/F 男/女	Nil 無	Non-vegetarian 非素食
109	Tai Po 大埔區	Tung Wah Group of Hospitals 東華三院	TWGHs Pao Siu Loong Care and Attention Home 東華三院包兆龍護理安老院@	Lot 152 in DD 27 (also known as 93 Sam Mun Tsai Road), Tai Po, New Territories 新界大埔丈量第27約地段第152號 (亦稱三門仔路93號)	26654011	26621736	C&A Home Providing COC	0	0	0	191	0	**191**	M/F 男/女	Nil 無	Non-vegetarian 非素食
110	Tai Po 大埔區	Tung Wah Group of Hospitals 東華三院	TWGHs Wu Chiang Wai Fong Care and Attention Home 東華三院伍蔣惠芳護理安老院@	Lot 152 in DD 27 (also known as 93 Sam Mun Tsai Road), Tai Po, New Territories 新界大埔丈量第27約地段第152號 (亦稱三門仔路93號)	26671234	26676203	C&A Home Providing COC	0	0	0	203	0	**203**	M/F 男/女	Nil 無	Non-vegetarian 非素食
111	Tai Po 大埔區	Caritas - Hong Kong 香港明愛	Caritas Fu Heng Home - Tai Po 明愛富亨苑†@	G/F & 1/F, Heng Wing House, Fu Heng Estate, Tai Po, New Territories 新界大埔富亨邨亨榮樓地下及1字樓	26606125	26656872	Conversion Home Providing COC	0	0	0	100	0	**100**	M/F 男/女	Catholic 天主教	Non-vegetarian 非素食
112	Tai Po 大埔區	Tsung Tsin Mission of Hong Kong Social Service (The) 基督教香港崇真會社會服務	Tsung Tsin Mission of Hong Kong Kwong Fuk Home for the Elderly 基督教香港崇真會廣福頤養院†	3/F, Kwong Yan House, Kwong Fuk Estate, Tai Po, New Territories 新界大埔廣福邨廣仁樓3字樓	26530636	26537932	Conversion Home Providing COC	0	0	0	100	0	**100**	M/F 男/女	Christian 基督教	Non-vegetarian 非素食
113	Tai Po 大埔區	Sik Sik Yuen 嗇色園	Ho Shin Home for the Elderly (Sponsored by Sik Sik Yuen) 嗇色園主辦可善護理安老院†@	G/F & 1/F, Shin King House, Fu Shin Estate, Tai Po, New Territories 新界大埔富善邨善景樓地下及二樓	26616555	26614772	Conversion Home Providing COC	0	0	0	90	0	**90**	M/F 男/女	Buddhist/ Taoist/ Confucian 佛/道/孔教	Non-vegetarian 非素食

List of Subvented, Self-financing and Contract Residential Care Homes for the Elderly Providing Subsidised Places for the Elderly (As at 30.6.2023)
提供資助安老服務宿位的津助院舍、自負盈虧院舍及合約院舍名單 (截至 30.6.2023)

S/N 編號	District 地區	Agency 機構	Name of Home 院舍名稱	Address 地址	Tel. 電話	Fax 傳真	Type of Service 服務類別	Type of Place 宿位種類: Hostel for the Elderly 長者宿舍	Home for the Aged 安老院	Care-and-Attention 護理安老院	Care-and-Attention Providing a Continuum-of-Care 提供持續照顧的護理安老院	Nursing Home 護養院	Total 總數	Sex 性別	Religion 宗教	Diet 膳食
114	North 北區	Tung Wah Group of Hospitals 東華三院	TWGHs Fong Wong Woon Tei Home for the Elderly 東華三院方王換娣安老院†	Wings A & B, G/F, and 1/F, Cheung Tak House, Cheung Wah Estate, Fanling, New Territories 新界粉嶺祥華邨祥德樓地下A及B翼及1字樓	26697697	26690265	Conversion Home Providing COC	0	0	0	75	0	**75**	M/F 男/女	Nil 無	Non-vegetarian 非素食
115	North 北區	Tung Wah Group of Hospitals 東華三院	TWGHs Po Chung Chuen Ying Home for the Elderly 東華三院寶鍾全英安老院†@	No.1, G/F & 1/F, Shun Ming House, Wah Ming Estate, Fanling, New Territories 新界粉嶺華明邨信明樓地下及1字樓1號	26759308	26762770	Conversion Home Providing COC	0	0	0	88	0	**88**	M/F 男/女	Nil 無	Non-vegetarian 非素食
116	North 北區	Heung Hoi Ching Kok Lin Association 香海正覺蓮社	Heung Hoi Ching Kok Lin Association Buddhist Po Ching Care and Attention Home for the Aged Women 香海正覺蓮社佛教寶靜護理安老院@	10 Chi Fuk Circuit, Fanling, New Territories 新界粉嶺置福圍10號	26695438	26690235	C&A Home Providing COC	0	0	0	128	0	**128**	F 女	Buddhist 佛教	Vegetarian 素食
117	North 北區	Heung Hoi Ching Kok Lin Association 香海正覺蓮社	Heung Hoi Ching Kok Lin Association Buddhist Li Chong Yuet Ming Nursing Home for the Elderly 香海正覺蓮社主辦佛教李莊月明護養院	5 Po Ping Road, Sheung Shui, New Territories 新界上水保平路5號	21450238	21450236	Nursing Home	0	0	0	0	254	**254**	M/F 男/女	Buddhist 佛教	Vegetarian/ Non-vegetarian 素食/ 非素食
118	North 北區	Heung Hoi Ching Kok Lin Association 香海正覺蓮社	Heung Hoi Ching Kok Lin Association Buddhist Po Ching Home for the Aged Women 香海正覺蓮社佛教寶靜安老院†@	8 Chi Fuk Circuit, Fanling, New Territories 新界粉嶺置福圍八號	26697123	26694092	Conversion Home Providing COC	0	0	0	270	0	**270**	F 女	Buddhist 佛教	Vegetarian 素食
119	North 北區	Chinese YMCA of Hong Kong 香港中華基督教青年會	Chinese YMCA of Hong Kong Tin Ping Care and Attention Home for the Elderly 香港中華基督教青年會天平頤康之家†	2/F, Tin Ming House, Tin Ping Estate, Sheung Shui, New Territories 新界上水天平邨天明樓二樓	26733781	26680246	Conversion Home Providing COC	0	0	0	79	0	**79**	M/F 男/女	Christian 基督教	Non-vegetarian 非素食
120	North 北區	Caritas - Hong Kong 香港明愛	Caritas Fung Wong Fung Ting Home 明愛馮黃鳳亭安老院@	DD 82, Lot 1562, Fanling, New Territories (also known as Ping Che Road, Ta Kwu Ling) 新界粉嶺丈量約份第82約地段第1562號（亦稱打鼓嶺坪輋路）	26592382	26747294	C&A Home Providing COC	0	0	0	120	0	**120**	M/F 男/女	Catholic 天主教	Non-vegetarian 非素食
121	North 北區	Women's Welfare Club (Eastern District) Hong Kong 香港東區婦女福利會	Women's Welfare Club (Eastern District) Hong Kong Wong Fung Ting Hostel for the Elderly (The) 香港東區婦女福利會黃鳳亭頤安苑†	4/F, Choi Chu House, Choi Yuen Estate, Sheung Shui, New Territories 新界上水彩園邨彩珠樓4字樓	26725032	26685060	Conversion Home Providing COC	0	0	0	65	0	**65**	F 女	Nil 無	Non-vegetarian 非素食
122	North 北區	Fung Kai Public School 鳳溪公立學校	Fung Kai Care and Attention Home for the Elderly - Home Section 鳳溪護理安老院 - 安老部†	G/F to 3/F, 22 Tin Ping Road (F.S.S.T.L. 174), Sheung Shui, New Territories 新界上水天平路22號（粉嶺上水地段第174號）地下至三字樓	26839800	26839850	Conversion Home Providing COC	0	0	0	100	0	**100**	M/F 男/女	Nil 無	Non-vegetarian 非素食
123	North 北區	Fung Kai Public School 鳳溪公立學校	Fung Kai Care and Attention Home for the Elderly - C & A Section 鳳溪護理安老院 - 護理部±	4/F-6/F, 22 Tin Ping Road (F.S.S.T.L. 174), Sheung Shui, New Territories 新界上水天平路22號（粉嶺上水地段第174號）4至6樓	26839700	26839740	NHPPS Home	0	0	0	0	45	**45**	M/F 男/女	Nil 無	Non-vegetarian 非素食
124	North 北區	Lucky Stable Limited 祥尊有限公司	Park Prime . Po Shek Wu 柏悅年華・寶石湖 *	Unit No. 1, 3/F-4/F, Po Shek Wu Estate Ancillary Facilities Block, 23 Choi Yuen Roard, Sheung Shui, New Territories 新界上水彩園路23號寶石湖邨服務設施大樓三樓至四樓一號舖	23229338	23229883	Contract Home	0	0	0	8	72	**80**	M/F 男/女	Nil 無	Non-vegetarian 非素食
125	Yuen Long 元朗區	Yan Oi Tong Limited 仁愛堂有限公司	Yan Oi Tong Tin Ka Ping Care and Attention Home 仁愛堂田家炳護理安老院†	G/F & 1/F, Wah Ping House, Long Ping Estate, Yuen Long, New Territories 新界元朗朗屏邨畫屏樓地下及二樓	24793360	24764605	Conversion Home Providing COC	0	0	0	88	0	**88**	M/F 男/女	Nil 無	Non-vegetarian 非素食
126	Yuen Long 元朗區	Tung Wah Group of Hospitals 東華三院	TWGHs Y. C. Liang Memorial Home for the Elderly 東華三院梁昌紀念安老院†@	G/F & 1/F, Yiu Yat House, Tin Yiu Estate, Tin Shui Wai, New Territories 新界天水圍天耀邨耀逸樓地下及1字樓	24451083	24453155	Conversion Home Providing COC	0	0	0	88	0	**88**	M/F 男/女	Nil 無	Non-vegetarian 非素食

List of Subvented, Self-financing and Contract Residential Care Homes for the Elderly Providing Subsidised Places for the Elderly (As at 30.6.2023)
提供資助安老服務宿位的津助院舍、自負盈虧院舍及合約院舍名單 (截至 30.6.2023)

S/N<br>編號	District<br>地區	Agency<br>機構	Name of Home<br>院舍名稱	Address<br>地址	Tel.<br>電話	Fax<br>傳真	Type of Service<br>服務類別	Type of Place 宿位種類<br>Hostel for the Elderly<br>長者宿舍	Home for the Aged<br>安老院	Care-and-Attention<br>護理安老院	Care-and-Attention Providing a Continuum-of-Care<br>提供持續照顧的護理安老院	Nursing Home<br>護養院	Total<br>總數	Sex<br>性別	Religion<br>宗教	Diet<br>膳食
127	Yuen Long<br>元朗區	Caritas - Hong Kong<br>香港明愛	Caritas Ying Shui Home<br>明愛盈水閣†@	3/F, Ying Shui House, Shui Pin Wai Estate, Yuen Long, New Territories<br>新界元朗水邊圍邨盈水樓三字樓	24797365	24421067	Conversion Home Providing COC	0	0	0	80	0	**80**	M/F<br>男/女	Catholic<br>天主教	Non-vegetarian<br>非素食
128	Yuen Long<br>元朗區	Salvation Army (The)<br>救世軍	Salvation Army Kam Tin Residence for Senior Citizens (The)<br>救世軍錦田長者之家@	103, Kam Tin Road, Yuen Long, New Territories<br>新界元朗錦田公路103號	29441369	29441638	C&A Home Providing COC	0	0	0	150	0	**150**	M/F<br>男/女	Christian<br>基督教	Non-vegetarian<br>非素食
129	Yuen Long<br>元朗區	Pok Oi Hospital<br>博愛醫院	Pok Oi Hospital Yeung Chun Pui Care and Attention Home<br>博愛醫院楊晉培護理安老院@	58 Sha Chau Lei Tsuen, Ha Tsuen, Yuen Long, New Territories<br>新界元朗廈村沙州里村58號	24721377	24722952	C&A Home Providing COC	0	0	0	143	0	**143**	M/F<br>男/女	Nil<br>無	Non-vegetarian<br>非素食
130	Yuen Long<br>元朗區	Pok Oi Hospital<br>博愛醫院	Pok Oi Hospital Tai Kwan Care & Attention Home<br>博愛醫院戴均護理安老院@	G/F-2/F & KW307, Shui Kwok House, Tin Shui Estate, Tin Shui Wai, Yuen Long, New Territories<br>新界元朗天水圍天瑞邨瑞國樓地下至2字樓及KW 307室	24473886	24482290	C&A Home Providing COC	0	0	0	109	0	**109**	M/F<br>男/女	Nil<br>無	Non-vegetarian<br>非素食
131	Yuen Long<br>元朗區	Pok Oi Hospital<br>博愛醫院	Pok Oi Hospital Jockey Club Care and Attention Home<br>博愛醫院賽馬會護理安老院@	Lot 1392 and Lot 837 R.P. in D.D. 115, Au Tau, Yuen Long, New Territories<br>新界元朗頭第115約第1392地段及837餘段	24702266	24700346	C&A Home Providing COC	0	0	0	165	0	**165**	M/F<br>男/女	Nil<br>無	Non-vegetarian<br>非素食
132	Yuen Long<br>元朗區	Pok Oi Hospital<br>博愛醫院	Pok Oi Hospital Centenary Chan See Memorial Nursing Home cum Day Care Centre<br>博愛醫院百周年陳是紀念護養院暨日間中心 *	2/F to 4F, 10 Kwong Yip Street, Yuen Long, New Territories<br>新界元朗擴業街10號二樓至四樓	27120998	27120977	Contract Home	0	0	0	7	68	**75**	M/F<br>男/女	Nil<br>無	Non-vegetarian<br>非素食
133	Yuen Long<br>元朗區	Ching Chung Taoist Association of Hong Kong Limited<br>青松觀有限公司	Ching Chung Taoist Association of Hong Kong Limited Ching Chung Care and Attention Home for the Aged<br>青松觀有限公司青松護理安老院@	DD125 Lot 2317 Ha Tsuen, Yuen Long, New Territories (also known as 57, Sha Chau Lei Chuen, Ping Ha Road, Yuen Long)<br>新界元朗廈村第125約第2317地段（亦稱元朗屏廈路沙州里村57號）	24721393	24721458	C&A Home Providing COC	0	0	0	120	0	**120**	M/F<br>男/女	Taoist<br>道教	Non-vegetarian<br>非素食
134	Yuen Long<br>元朗區	Po Leung Kuk<br>保良局	Po Leung Kuk Tin Yan Home for the Elderly cum Green Joy Day Care Centre for the Elderly<br>保良局天恩護老院暨耆昌長者日間護理中心*	3/F and 4/F, Ancillary Facilities Block, Tin Yan Estate, Tin Shui Wai, New Territories<br>新界天水圍天恩邨服務設施大樓三樓及四樓	37410850	37410851	Contract Home	0	0	0	7	67	**74**	M/F<br>男/女	Nil<br>無	Non-vegetarian<br>非素食
135	Tuen Mun<br>屯門區	Yan Chai Hospital<br>仁濟醫院	Yan Chai Hospital Tsin Man Kuen Elderly Home<br>仁濟醫院錢曼娟安老院†	1/F, Oi Lok House, Yau Oi Estate, Tuen Mun, New Territories<br>新界屯門友愛邨愛樂樓二樓	24512323	24526976	Conversion Home Providing COC	0	0	0	59	0	**59**	M/F<br>男/女	Nil<br>無	Non-vegetarian<br>非素食
136	Tuen Mun<br>屯門區	Tung Wah Group of Hospitals<br>東華三院	TWGHs Tai Tung Pui Care & Attention Home<br>東華三院戴東培護理安老院@	1/F to 4/F, TWGHs Tai Tung Pui Social Service Building, 32 Tsing Sin Street, Tuen Mun, New Territories<br>新界屯門青善街32號東華三院戴東培社會服務大樓1字樓至4字樓	24508461	24578916	C&A Home Providing COC	0	0	0	204	0	**204**	M/F<br>男/女	Nil<br>無	Non-vegetarian<br>非素食
137	Tuen Mun<br>屯門區	Caritas - Hong Kong<br>香港明愛	Caritas Li Ka Shing Care and Attention Home<br>明愛李嘉誠護理安老院@	16 Wah Fat Street, Tuen Mun, New Territories<br>新界屯門華發街16號	24412923	24414669	C&A Home Providing COC	0	0	0	260	0	**260**	M/F<br>男/女	Catholic<br>天主教	Non-vegetarian<br>非素食
138	Tuen Mun<br>屯門區	Kiangsu Chekiang and Shanghai Residents (Hong Kong) Association<br>香港蘇浙滬同鄉會	Kiangsu Chekiang and Shanghai Residents (Hong Kong) Association Tuen Mun Hostel for the Elderly<br>香港蘇浙滬同鄉會屯門安老院†	Units 223-234 & Units 321-368, Tip Sum House, Butterfly Estate, Tuen Mun, New Territories<br>新界屯門蝴蝶邨蝶心樓223至234室及321至368室	24675967	24648974	Conversion Home Providing COC	0	0	0	117	0	**117**	M/F<br>男/女	Nil<br>無	Non-vegetarian<br>非素食
139	Tuen Mun<br>屯門區	Evangelical Lutheran Church of Hong Kong (The)<br>基督教香港信義會	The Evangelical Lutheran Church of Hong Kong Shan King Care and Attention Home for the Elderly<br>基督教香港信義會山景長者護理院@	1/F and 2/F (part of), King Wing House, Shan King Estate, Tuen Mun, New Territories<br>新界屯門山景邨景榮樓二樓及三樓 （部分）	24676612	24636689	C&A Home Providing COC	0	0	0	150	0	**150**	M/F<br>男/女	Christian<br>基督教	Non-vegetarian<br>非素食

List of Subvented, Self-financing and Contract Residential Care Homes for the Elderly Providing Subsidised Places for the Elderly (As at 30.6.2023)
提供資助安老服務宿位的津助院舍、自負盈虧院舍及合約院舍名單 (截至 30.6.2023)

S/N 編號	District 地區	Agency 機構	Name of Home 院舍名稱	Address 地址	Tel. 電話	Fax 傳真	Type of Service 服務類別	Type of Place 宿位種類					Total 總數	Sex 性別	Religion 宗教	Diet 膳食
								Hostel for the Elderly 長者宿舍	Home for the Aged 安老院	Care-and-Attention 護理安老院	Care-and-Attention Providing a Continuum-of-Care 提供持續照顧的護理安老院	Nursing Home 護養院				
140	Tuen Mun 屯門區	Pok Oi Hospital 博愛醫院	Pok Oi Hospital Tuen Mun Nursing Home 博愛醫院屯門護養院	2 Siu Lun Street, Tuen Mun, New Territories 新界屯門兆麟街2號	24578123	24582723	Nursing Home	0	0	0	0	216	**216**	M/F 男/女	Nil 無	Non-vegetarian 非素食
141	Tuen Mun 屯門區	Sik Sik Yuen 嗇色園	Ho Cheung Home for the Elderly (Sponsored by Sik Sik Yuen) 嗇色園主辦可祥護理安老院†@	G/F and 1/F, Leung Wah House, Leung King Estate, Tuen Mun, New Territories 新界屯門良景邨良華樓地下及1字樓	24668677	24667531	Conversion Home Providing COC	0	0	0	90	0	**90**	M/F 男/女	Buddhist/ Taoist/ Confucian 佛/道/孔教	Non-vegetarian 非素食
142	Tuen Mun 屯門區	Ching Chung Taoist Association of Hong Kong Limited 青松觀有限公司	Ching Chung Home for the Aged 青松安老院†	G/F, 1/F and 2/F, Man Ching House, 8 Tsing Chung Path, Tuen Mun, New Territories 新界屯門青松徑8號晚晴樓地下、二樓及三樓	24611010	24603099	Conversion Home Providing COC	0	0	0	60	0	**60**	M/F 男/女	Taoist 道教	Non-vegetarian 非素食
143	Tuen Mun 屯門區	Miu Fat Buddhist Monastery 妙法寺	MFBM Elderly Home 妙法寺護老院±	L1-L5, Dormitory Block, Lot No. 2821 in DD 130, Tuen Mun, New Territories (also known as L1-L5, Dormitory Block, 18 Castle Peak Road, Lam Tei) 新界屯門丈量約份第130約地段第2821號院舍座L1-L5（亦稱藍地青山公路18號院舍座L1-L5）	35951008	83431699	NHPPS Home	0	0	0	0	48	**48**	F 女	Buddhist 佛教	Vegetarian 素食
144	Tuen Mun 屯門區	Hong Kong Baptist Mr & Mrs Au Shue Hung Rehabilitation and Healthcare Home Limited 香港浸信會區樹洪伉儷康復護養院有限公司	The Greenville Care Home 青怡居*	B/F (portion), G/F (portion), 1/F (portion), 2/F (portion), 3/F (portion) and 5/F (portion), 2 Tsing Min Path, Tuen Mun, New Territories 新界屯門青棉徑2號地庫（部分）、地下（部分）、一樓(部分）、二樓(部分）、三樓(部分）及五樓(部分）	36195515	36195533	Contract Home	0	0	0	6	54	**60**	M/F 男/女	Nil 無	Non-vegetarian 非素食
145	Tsuen Wan 荃灣區	Yan Chai Hospital 仁濟醫院	Yan Chai Nursing Home 仁濟護養院	4/F-8/F, Yan Chai Hospital Multi-Services Complex, 18 Yan Chai Street, Tsuen Wan, New Territories 新界荃灣仁濟街18號仁濟醫院綜合服務大樓四至八樓	24092888	24095888	Nursing Home	0	0	0	0	316	**316**	M/F 男/女	Nil 無	Non-vegetarian 非素食
146	Tsuen Wan 荃灣區	Tung Lum Nien Fah Tong Limited 東林念佛堂有限公司	Tung Lum Buddhist Aged Home 佛教東林安老院†@	29 Tung Lam Terrace, Fu Yung Shan, Tsuen Wan, New Territories 新界荃灣芙蓉山東林台29號	24982558	24906969	Conversion Home Providing COC	0	0	0	70	0	**70**	M 男	Buddhist 佛教	Vegetarian 素食
147	Tsuen Wan 荃灣區	Hong Kong Society for the Aged (The) 香港耆康老人福利會	SAGE Shek Wai Kok Home for the Elderly 耆康會石圍角護理安老院†@	Units 201-260, Shek Lin House, Shek Wai Kok Estate, Tsuen Wan, New Territories 新界荃灣石圍角邨石蓮樓201-260室	24980423	24990964	Conversion Home Providing COC	0	0	0	93	0	**93**	M/F 男/女	Nil 無	Non-vegetarian 非素食
148	Tsuen Wan 荃灣區	Hong Kong Bodhi Siksa Society Limited (The) 香港菩提學會有限公司	Hong Kong Bodhi Siksa Society, Ltd. Care and Attention Home for the Aged (The) 香港菩提學會主辦佛教菩提護理安老院@	38 Lo Wai Road, Tsuen Wan, New Territories 新界荃灣老圍路38號	24022240	24177301	C&A Home Providing COC	0	0	0	150	0	**150**	M/F 男/女	Buddhist 佛教	Vegetarian/ Non-vegetarian 素食/ 非素食
149	Tsuen Wan 荃灣區	Yuen Yuen Institute (The) 圓玄學院	Yuen Yuen Home for the Aged 圓玄安老院†	31 Lo Wai Road, Tsuen Wan, New Territories 新界荃灣老圍路31號	24992818	24153626	Conversion Home Providing COC	0	0	0	55	0	**55**	M/F 男/女	Buddhist/ Taoist/ Confucian 佛/道/孔教	Non-vegetarian 非素食
150	Tsuen Wan 荃灣區	Yuen Yuen Institute (The) 圓玄學院	Yuen Yuen Care & Attention Home for the Aged 圓玄護理安老院@	33 Lo Wai Road, Tsuen Wan, New Territories 新界荃灣老圍路33號	24021515	24021502	C&A Home Providing COC	0	0	0	150	0	**150**	M/F 男/女	Buddhist/ Taoist/ Confucian 佛/道/孔教	Non-vegetarian 非素食

List of Subvented, Self-financing and Contract Residential Care Homes for the Elderly Providing Subsidised Places for the Elderly (As at 30.6.2023)
提供資助安老服務宿位的津助院舍、自負盈虧院舍及合約院舍名單 (截至 30.6.2023)

S/N 編號	District 地區	Agency 機構	Name of Home 院舍名稱	Address 地址	Tel. 電話	Fax 傳真	Type of Service 服務類別	Type of Place 宿位種類: Hostel for the Elderly 長者宿舍	Home for the Aged 安老院	Care-and-Attention 護理安老院	Care-and-Attention Providing a Continuum-of-Care 提供持續照顧的護理安老院	Nursing Home 護養院	Total 總數	Sex 性別	Religion 宗教	Diet 膳食
151	Tsuen Wan 荃灣區	Yuen Yuen Institute (The) 圓玄學院	Yuen Yuen Nursing Home cum Day Care Centre for the Elderly (Lei Muk Shue Estate) 圓玄護養院暨長者日間護理中心(梨木樹邨)*	1/F-3/F, Hong Shue House, Lei Muk Shue Estate, Tsuen Wan, New Territories 新界荃灣梨木樹邨康樹樓1至3字樓	24221681	24223661	Contract Home	0	0	0	7	72	**79**	M/F 男/女	Buddhist/ Taoist/ Confucian 佛/道/孔教	Non-vegetarian 非素食
152	Tsuen Wan 荃灣區	E.T. Investment Limited 頤盈投資有限公司	Olive Nursing Home cum Day Care Unit for the Elderly 紫雲間雋逸護養院暨長者日間護理單位*	G/F and 2/F (portion), 48 Wing Shun Street, Tsuen Wan, New Territories 新界荃灣永順街48號地下及2樓（部分）	35202133	35202131	Contract Home	0	0	0	10	86	**96**	M/F 男/女	Nil 無	Non-vegetarian 非素食
153	Tsuen Wan 荃灣區	Caritas - Hong Kong 香港明愛	Caritas Jockey Club Everbright Home 明愛賽馬會恩暉苑	G/F (portion), 1/F (portion), 4/F (portion) and 5/F-7/F, Caritas Jockey Club Tsuen Wan Social Service Building, 9 Shing Mun Road, Tsuen Wan, New Territories 新界荃灣城門道9號明愛賽馬會荃灣服務樓地下（部分）、1字樓（部分）、4字樓（部分）及5字樓至7字樓	37072046	36184480	C&A Home Providing COC	0	0	0	88	0	**88**	M/F 男/女	Catholic 天主教	Non-vegetarian 非素食
154	Kwai Tsing 葵青區	The Chinese Rhenish Church Hong Kong Synod 中華基督教禮賢會香港區會	Sze Tian Rhenish Home for the Elderly 禮賢會詩田頤養院†	5/F, Block 5, Kwai Shing West Estate, Kwai Chung, New Territories 新界葵涌葵盛西邨第5座5字樓	24272671	24191927	Conversion Home Providing COC	0	0	0	84	0	**84**	M/F 男/女	Christian 基督教	Non-vegetarian 非素食
155	Kwai Tsing 葵青區	The Chinese Rhenish Church, Hong Kong Synod 中華基督教禮賢會香港區會	Kwai Shing East Rhenish Care and Attention Home 禮賢會葵盛東護理安老院	1/F and Wing B and C of G/F, Shing Lok House and Shing Fu House, Kwai Shing East Estate, Kwai Chung, New Territories 新界葵涌葵盛東邨盛樂樓及盛富樓1字樓及地下B、C翼	24396644	24061380	C&A Home Providing COC	0	0	0	150	0	**150**	M/F 男/女	Christian 基督教	Non-vegetarian 非素食
156	Kwai Tsing 葵青區	Yan Chai Hospital 仁濟醫院	Yan Chai Hospital Mrs Kwok Yuk Cheung Care & Attention Home 仁濟醫院郭玉章夫人護理安老院@	33 Lai Chi Ling Road, Kwai Chung, New Territories 新界葵涌荔枝嶺路33號	27858723	27860670	C&A Home Providing COC	0	0	0	246	0	**246**	M/F 男/女	Nil 無	Non-vegetarian 非素食
157	Kwai Tsing 葵青區	Yan Chai Hospital 仁濟醫院	Yan Chai Hospital Hong Kong Peninsula Lions Club Elderly Home 仁濟醫院香港半島獅子會安老院†	Wing A and C on 3/F, Wing A, B and C on 4/F, Hong Shun House, Cheung Hong Estate, Tsing Yi, New Territories 新界青衣長康邨康順樓3字樓A、C翼及4字樓A、B、C翼	24337877	24979547	Conversion Home Providing COC	0	0	0	94	0	**94**	M/F 男/女	Nil 無	Non-vegetarian 非素食
158	Kwai Tsing 葵青區	Yan Chai Hospital 仁濟醫院	Yan Chai Hospital Artiste Training Alumni Association Care and Attention Home 仁濟醫院藝進同學會護理安老院@	G/F & 1/F, Shek Wah House and Shek Kai House, Shek Lei (II) Estate, Kwai Chung, New Territories 新界葵涌石籬（二）邨石華樓及石佳樓地下及1字樓	24803323	24803831	C&A Home Providing COC	0	0	0	155	0	**155**	M/F 男/女	Nil 無	Non-vegetarian 非素食
159	Kwai Tsing 葵青區	Yan Chai Hospital 仁濟醫院	Yan Chai Hospital Chinachem Care & Attention Home 仁濟醫院華懋護理安老院@	35, Lai Chi Ling Road, Kwai Chung, New Territories 新界葵涌荔枝嶺路35號	23713883	23713282	C&A Home Providing COC	0	0	0	278	0	**278**	M/F 男/女	Nil 無	Non-vegetarian 非素食
160	Kwai Tsing 葵青區	Helping Hand 伸手助人協會	Helping Hand Vera R. Desai Lai Yiu Care Home 伸手助人協會維拉荻茜麗瑤護老院	G/F (portion), 1/F and 2/F, Block B, Lai Yiu Shopping Centre, Lai Yiu Estate, Kwai Chung, New Territories 新界葵涌麗瑤邨麗瑤商場大廈B座地下（部分）、1字樓及2字樓	27852127	27424645	C&A Home Providing COC	0	0	0	76	0	**76**	M/F 男/女	Nil 無	Non-vegetarian 非素食
161	Kwai Tsing 葵青區	Hong Kong Christian Service 香港基督教服務處	Hong Kong Christian Service Cheung Fat Home for the Elderly 香港基督教服務處長發安老院†	G/F & 1/F, Leung Fat House, Cheung Fat Estate, Tsing Yi, New Territories 新界青衣長發邨亮發樓地下及2樓	24340143	24347337	Conversion Home Providing COC	0	0	0	80	0	**80**	M/F 男/女	Christian 基督教	Non-vegetarian 非素食

List of Subvented, Self-financing and Contract Residential Care Homes for the Elderly Providing Subsidised Places for the Elderly (As at 30.6.2023)
提供資助安老服務宿位的津助院舍、自負盈虧院舍及合約院舍名單 (截至 30.6.2023)

S/N 編號	District 地區	Agency 機構	Name of Home 院舍名稱	Address 地址	Tel. 電話	Fax 傳真	Type of Service 服務類別	Type of Place 宿位種類					Total 總數	Sex 性別	Religion 宗教	Diet 膳食
								**Hostel for the Elderly** 長者宿舍	**Home for the Aged** 安老院	**Care-and-Attention** 護理安老院	**Care-and-Attention Providing a Continuum-of-Care** 提供持續照顧的護理安老院	**Nursing Home** 護養院				
162	Kwai Tsing 葵青區	Hong Kong Sheng Kung Hui Welfare Council Limited 香港聖公會福利協會有限公司	Hong Kong Sheng Kung Hui Lam Woo Home for the Elderly 香港聖公會林護長者之家@	G/F and 1/F, Fung Yam House and Shing Yam House, On Yam Estate, Kwai Chung, New Territories 新界葵涌安蔭邨豐蔭樓及盛蔭樓地下及一樓	24010212	24284427	C&A Home Providing COC	0	0	0	157	0	**157**	M/F 男/女	Christian 基督教	Non-vegetarian 非素食
163	Kwai Tsing 葵青區	Hong Kong Lutheran Social Service, the Lutheran Church Hong Kong Synod Limited 香港路德會社會服務處	Hong Kong Lutheran Social Service Mrs Leung Kwai Yee Lutheran Home for the Elderly 香港路德會社會服務處路德會梁季彝夫人安老院†@	Units 101-108, 117-124 on G/F and Units 201-224 on 2/F, On Mei House, Cheung On Estate, Tsing Yi, New Territories 新界青衣長安邨安湄樓地下101-108號、117-124號及二樓201-224號	24976213	24325508	Conversion Home Providing COC	0	0	0	104	0	**104**	M/F 男/女	Christian 基督教	Non-vegetarian 非素食
164	Kwai Tsing 葵青區	Kiangsu Chekiang and Shanghai Residents (Hong Kong) Association 香港蘇浙滬同鄉會	Kiangsu Chekiang and Shanghai Residents (Hong Kong) Association Kwai Tsing Hostel for the Elderly 香港蘇浙滬同鄉會葵青良友安老院†	G/F and 1/F, Fu Kwok House, Tai Wo Hau Estate, Tsuen Wan, New Territories 新界荃灣大窩口邨富國樓地下及二樓	26143825	24197763	Conversion Home Providing COC	0	0	0	84	0	**84**	M/F 男/女	Nil 無	Non-vegetarian 非素食
165	Kwai Tsing 葵青區	Sik Sik Yuen 嗇色園	Ho On Home for the Elderly (Sponsored by Sik Sik Yuen) 嗇色園主辦可安護理安老院†	2/F, Shek Ning House, Shek Lei (I) Estate, Kwai Chung, New Territories 新界葵涌石籬（一）邨石寧樓二樓	24213038	24217090	Conversion Home Providing COC	0	0	0	78	0	**78**	M/F 男/女	Buddhist/ Taoist/ Confucian 佛/道/孔教	Non-vegetarian 非素食
166	Kwai Tsing 葵青區	Chung Sing Benevolent Society 鐘聲慈善社	Chung Sing Benevolent Society Lau Mui Hin Home for the Elderly 鐘聲慈善社劉梅軒安老院†@	Wing A and B of G/F and Wings A, B and C of 2/F, Yee Yat House, Tsing Yi Estate, New Territories 新界青衣邨宜逸樓地下A、B翼及2字樓A、B及C翼	24339881	24362672	Conversion Home Providing COC	0	0	0	83	0	**83**	M/F 男/女	Nil 無	Non-vegetarian 非素食
167	Kwai Tsing 葵青區	Po Leung Kuk 保良局	Po Leung Kuk Comfort Court for the Senior cum Evergreen Day Care Centre for the Elderly 保良局樂安居暨耆盛長者日間護理中心*	G/F (part) and 2/F (part) to 7/F, Shing Wo House, Kwai Shing Estate, Kwai Chung, New Territories 新界葵涌葵盛東邨盛和樓地下（部分）及二樓（部分）至七樓	35202770	35202771	Contract Home	0	0	0	15	129	**144**	M/F 男/女	Nil 無	Non-vegetarian 非素食
168	Kwai Tsing 葵青區	Charmind Limited 卓金有限公司	Evergreen Nursing Home cum Day Care Centre 松悅園耆欣護養院暨日間護理中心*	LG/F (part), G/F (part), 1/F - 3/F, Lai Shek House, Shek Yam Estate, Kwai Chung, New Territories 新界葵涌石蔭邨禮石樓低層地下（部分）、地下（部分）及一樓至三樓	26190881	26190700	Contract Home	0	0	0	8	75	**83**	M/F 男/女	Nil 無	Non-vegetarian 非素食
169	Kwai Tsing 葵青區	Evangelical Lutheran Church Social Service – Hong Kong 基督教香港信義會社會服務部	ELCHK, Grace Court 基督教香港信義會恩頤居±	2/F (North), 3/F, 4/F and 7/F (part), Fung Yat Social Service Complex, 364 Kwai Shing Circuit, Kwai Chung, New Territories 新界葵涌葵盛圍364號馮鎰社會服務大樓2樓（北座）、3樓、4樓及七樓（部分）	21552828	21552829	NHPPS Home	0	0	0	0	72	**72**	M/F 男/女	Christian 基督教	Non-vegetarian 非素食
170	Kwai Tsing 葵青區	Wai Ying Investment Limited 維盈投資有限公司	Evergreen (Kwai Chung Estate) Nursing Home 松悅園耆和護養院*	Unit 2, Podium Level 3, Kwai Chung Shopping Centre, Kwai Chung Estate, New Territories. 新界葵涌邨葵涌商場第三層平台2號	22794692	22794693	Contract Home	0	0	0	7	71	**78**	M/F 男/女	Nil 無	Non-vegetarian 非素食
171	Kwai Tsing 葵青區	Azure Elderly Care Limited 蔚耆苑有限公司	Aura Nursing Home cum Day Care Centre 耀耆頤養院暨日間中心*	G/F (portion) and Room 103, Podium 1/F, Kwai Tsui Estate, Kwai Chung, New Territories 新界葵涌葵翠邨地下（部分）及平台一層103室	28893551	28893557	Contract Home	0	0	0	7	63	**70**	M/F 男/女	Nil 無	Non-vegetarian 非素食
		**No. of Homes** 院舍數目	**171**				**Total No. of Places** 宿位總數	**0**	**9**	**71**	**15,480**	**4,651**	**20,211**			

List of Subvented, Self-financing and Contract Residential Care Homes for the Elderly Providing Subsidised Places for the Elderly (As at 30.6.2023)
提供資助安老服務宿位的津助院舍、自負盈虧院舍及合約院舍名單 (截至 30.6.2023)

S/N 編號	District 地區	Agency 機構	Name of Home 院舍名稱	Address 地址	Tel. 電話	Fax 傳真	Type of Service 服務類別	Type of Place 宿位種類					Total 總數	Sex 性別	Religion 宗教	Diet 膳食
								Hostel for the Elderly 長者宿舍	Home for the Aged 安老院	Care-and-Attention 護理安老院	Care-and-Attention Providing a Continuum-of-Care 提供持續照顧的護理安老院	Nursing Home 護養院				

Remarks:

*** These are contract homes.**
這些是合約安老院舍。

@ These Homes provide places under agency quota (AQ)
這些津助安老院舍提供「機構名額」宿位。

† For these homes, Care-and-Attention places with a Continuum-of-Care are to be created by phases and their existing hostel for the elderly and home for the aged places will eventually be fully phased out.
這些院舍的提供持續照顧的護理安老宿位會分階段產生，而其現有的長者宿舍和安老院宿位亦會分階段取消。

± These are self-financing homes participating in the Nursing Home Place Purchase Scheme (NHPPS). The subsidised nursing home places are created by phases with effect from December 2010.
這些是參與「護養院宿位買位計劃」的自負盈虧院舍。有關的資助護養院宿位由二零一零年十二月起分階段投入服務。

# The Home has decided to fade out from Nursing Home Place Purchase Scheme with cessation of new admission with effect from 1 April 2015 .
該院已決定淡出「護養院宿位買位計劃」，並於2015年4月1日起停止接收新的入住個案。

&The home is undergoing major renovation.
該院舍現正安排進行大型裝修工程。

Note 1:

All residential places of these homes will be phased out in the Conversion Programme. The Social Welfare Department has ceased to accept applications for admission to these homes.
這些院舍正分階段轉型，其宿位將逐步減少至全部取消。社會福利署已停止接受入住這些安老院舍的申請。

Note 2:

All subvented and contract homes provide 24-hour residential care services for elders. Office operating hours of these homes are normally from 9:00 a.m. to 5:00 p.m. from Mondays to Fridays, and from 9:00 a.m. to 12:00 p.m. on Saturdays (The actual operating hours of individual home may vary).
所有津助院舍及合約安老院舍均提供24小時長者住宿照顧服務。院舍的辦公室開放時間一般為星期一至五上午九時至下午五時，及星期六上午九時至中午十二時（個別院舍的開放時間或略有不同）。
